# RNA and protein immunization with *Trypanosoma cruzi* trans-sialidase containing SAPA repeats protects mice against infection and promotes a balanced inflammatory response

**DOI:** 10.3389/fcimb.2025.1681807

**Published:** 2025-10-17

**Authors:** Nailma Silva Aprigio dos Santos, Carlos Roberto de Almeida-Júnior, Mayra Fernanda Ricci, Rodrigo C. O. Sanches, Renata Salgado Fernandes, Gabriela de A. Burle-Caldas, Júlia Teixeira de Castro, João Luís Reis-Cunha, Daniella C. Bartholomeu, Ana Clara Martins Meira, Thaiane Gomes Nascimento, Natalia Fernanda de Melo Oliveira, Ricardo T. Gazzinelli, Fabiana S. Machado, Santuza M. R. Teixeira

**Affiliations:** 1 Departamento de Bioquímica e Imunologia, Universidade Federal de Minas Gerais, Belo Horizonte, Brazil; 2 Centro de Tecnologia de Vacinas, Universidade Federal de Minas Gerais, Belo Horizonte, Brazil; 3 Department of Biology and York Biomedical Research Institute, University of York, York, United Kingdom; 4 Departamento de Parasitologia, Universidade Federal de Minas Gerais, Belo Horizonte, Brazil

**Keywords:** Chagas disease, trans-sialidase, SAPA repeats, RNA, LNP, vaccine

## Abstract

Proteins with repeat domains are commonly found in protozoan parasites. *Trypanosoma cruzi*, which causes Chagas disease (CD), possesses a group of surface proteins called trans-sialidases (TS). These proteins are responsible for transferring sialic acid from the host’s glycoconjugates to the parasite’s mucins. The TS proteins feature a C-terminal immunogenic domain that includes amino acid repeats known as SAPA (Shed Acute Phase Antigen). Shed in the blood of the infected host, TS mediates several biological effects and because of its essential role during infection, it has been tested recurrently as a vaccine candidate against CD. Here, we investigate the effect of immunizing mice with recombinant TS proteins with and without (w/o) SAPA repeats, as well as with a protein containing only the repeat domain. We also immunize mice with RNA formulations encoding TS sequences with and without SAPA. Besides confirming the immunodominance of the SAPA domain, after challenging immunized animals with *T. cruzi*, we showed that the presence of the repeats did not significantly impact protection and parasite numbers after infection. However, immunization with TS protein or RNA containing the repeat domain resulted in increased production of IL-10 compared to mice immunized with TS without SAPA, and this increased IL-10 production correlates with a significant reduction in the inflammatory infiltrate in heart tissues of infected animals. These results indicate that the immunodominant SAPA domain plays a role in promoting an anti-inflammatory response, which, as a vaccine component, may contribute to induce a desirable, more balanced immune response.

## 1 Introduction

*Trypanosoma cruzi*, the etiologic agent of Chagas disease (CD), is a protozoan parasite whose life cycle alternates between invertebrate hosts belonging to a group of triatomine insects and several species of mammals including men. First described in 1909 by the physician Carlos Chagas, despite almost 120 years of intensive studies, Chagas disease continues to be a significant health issue, impacting 6–7 million individuals globally and resulting in around 50,000 fatalities annually, with no vaccines currently available. Although it is primarily endemic to Latin America, the disease has spread to other continents due to migration, with recent estimates suggesting that about 75 million people worldwide are at risk of contracting the infection (Urbina and Docampo, 2003; Rassi and Marin-Neto, 2010; Lidani et al., 2019). In a few countries where the disease is endemic, CD is still mainly spread to humans through triatomine vectors. However, in numerous regions, non-vectorial transmission methods such as organ transplants, blood transfusions, oral infection from contaminated food, and congenital transmission continue to pose a significant risk (Rassi and Marin-Neto, 2010). During vector transmission, metacyclic trypomastigote forms present in the feces and urine of an infected vector are deposited at the site of the insect bite and can be inoculated into the mammal bloodstream. The highly infective trypomastigote forms can infect any nucleated mammalian cell and after transforming into amastigotes they multiply by binary fission in the cytoplasm. Following multiple cycles of replication, amastigotes transform into non-replicating, flagellated trypomastigotes. These are then released into the bloodstream when the host cell undergoes lysis, allowing them to either infect additional cells or be ingested by another insect. After being ingested with a blood meal, trypomastigotes differentiate into epimastigotes within the insect intestinal tract where they replicate until reaching the posterior end of the intestine and differentiate into metacyclic, infective trypomastigotes (de Souza et al., 2010; Fernandes and Andrews, 2012). After infection, an acute phase characterized by high parasitemia and lasting about 2 months is followed by a chronic infection that is asymptomatic in most cases and may last for life. During the chronic phase, although parasitemia is undetectable, parasite persistence in the tissues may result in an indeterminate, asymptomatic form of CD, which occurs in most infected individuals or, in approximately 30% of cases, in the symptomatic form of CD, with cardiac, digestive or both clinical alterations (Cançado, 1999; Rassi and Marin-Neto, 2010). Two drugs used for treatment, benznidazole and nifurtimox, are effective only in the acute phase but are less efficient in the chronic phase of the disease, with adverse reactions that are more frequent at older age (Menna-Barreto and de Castro, 2017; Kratz et al., 2018). Considered a valuable strategy for preventing the disease or to minimize its complications, despite many efforts, no vaccine for Chagas disease has yet reached clinical trials (Teixeira et al., 2025). Most efforts towards the development of an effective vaccine against CD have been focused on surface parasite antigens, including members of the large family of trans-sialidase, Tc24 flagellar proteins and cruzipain, member of the cysteine protease family (Dumonteil and Herrera, 2021). These studies have uncovered the central role of interferon-gamma (IFN-γ) and the activation of CD8+ T cells as well as a balanced Th1/Th2 response to provide significant protection. However, a better understanding of the complex mechanisms developed by the parasite to evade the host immune response and the immunomodulation required to prevent tissue damage remain as major challenges still faced by researchers working on vaccine development for CD. One of the main features of protozoa parasites is the presence of proteins enriched in repetitive amino acid domains that are often targets of B-cell responses. In addition of being associated with protein–protein interaction processes required for host cell invasion, repeat-enriched proteins may be part of the parasite weaponry used for immune evasion. A study comparing the predicted proteomes of 27 protists, including intracellular and extracellular parasites as well as free-living organisms showed a bias in the repetitive content in the proteomes of obligate intracellular protozoan parasites including *T. cruzi* and different species of *Plasmodium* and *Leishmania* (Mendes et al., 2013). Parasite antigens, characterized by sequences predicted to be intrinsically disordered and a skewed amino acid composition, tend to have fewer predicted MHC I and II peptides but a higher number of predicted B-cell epitopes. It has been suggested that these repetitive domains might hinder an effective immune response by crosslinking B-cell receptors, thereby triggering a less effective T-cell independent immune response (Schofield, 1991). Previous studies from our group and others have shown that *T. cruzi* express different types of proteins containing repeat domains, including surface proteins, ribosomal protein, and flagellar proteins, which do not seem to share functional characteristics (DaRocha et al., 2002; Goto et al., 2008).

Trans-sialidases (TS) are among the *T. cruzi* surface proteins that contain a repeat domain known to elicit a strong humoral response early during infection. The TS sequences, encoded by the largest gene family in the *T. cruzi* genome with over 1,000 copies per genome were divided into eight distinct groups according to the presence of specific motifs (Freitas et al., 2011). Only members from group I TS contain sequences encoding TS proteins with a catalytic site, which contains a conserved tyrosine (Tyr342) located in the active site floor and involved in the formation of a covalent glucosyl-enzyme intermediate (Cremona et al., 1995; Amaya et al., 2004; Freitas et al., 2011; Oppezzo et al., 2011). Active TS transfers sialic acid (SA) residues from host glycoconjugates to mucins on the surface of the parasite, as this parasite is unable to synthesize these molecules (Dc-Rubin and Schenkman, 2012). Like *T. cruzi*, various pathogens have evolved to use SA to mediate binding to host cells or by coating themselves to evade host immune responses (Varki, 2008). Using CRISPR genome editing, our group showed that *T. cruzi* cannot survive in mammalian hosts without TS activity (Burle-Caldas et al., 2022). An important feature of some active TS is the presence of tandem repeats of 12 amino acids in the C-terminal portion of the protein, known as SAPA (shed acute phase antigen), that consists of the amino acids (DSSAHS/GTPSTPV/A) (Affranchino et al., 1989; Freitas et al., 2011). Shed in the bloodstream, TS activity mediates several biological effects including the induction of an immunosuppressive state during the acute phase of the infection (Freire-de-Lima et al., 2010). It has been shown that the SAPA repeats present in active TS increase the stability of the enzyme in the blood (Buscaglia et al., 1999). In addition to its role related to enzyme stability, the TS SAPA domain acts as a T-independent polyclonal activator of B cells, which is part of the mechanisms developed by the parasite to divert the immune system from important targets and to delay a more effective immune response (Gao et al., 2002). Various tests of a CD vaccine employing recombinant TS protein as a target antigen have shown promising results (Fontanella et al., 2008; Giddings et al., 2010; Bontempi et al., 2015; Pereira et al., 2015; Bontempi et al., 2017; da Costa et al., 2021; Gamba et al., 2021; Pacini et al., 2024). Because it belongs to a multigene family, different members of the TS family have been tested as vaccine candidates. However, in none of these studies the role of the TS SAPA domain has been addressed. Given the prevailing assumption that this repeat domain may be detrimental as a vaccine component, we decided to compare the protective effect of TS sequences with and without SAPA repeats. Since more recently, the use of RNA vaccines has gained prominence due to their ability to induce a strong cellular response, which is required for efficient protection against intracellular pathogens (Verbeke et al., 2022), we also compared immunization protocols based on RNA and recombinant proteins. In addition of promoting increased protection, testing an RNA vaccine platform will also allow us, in future experiments, to combine different sequences in one vaccine formulation, which may be highly beneficial for a *T. cruzi* antigen that presents such a large sequence heterogeneity in the parasite population. Using both vaccine platforms, we investigate the role played by SAPA repeat domain of TS during *T. cruzi* infection and its influence as a component of a vaccine for CD by evaluating the effect of immunizing mice with TS sequences with or without SAPA repeats before challenging with a virulent *T. cruzi* strain.

## 2 Material and methods

### 2.1 Ethics statement

This study was performed with the approval of the Ethics Committee on the Use of Animals (CONCEA) from the Federal University of Minas Gerais (permit number 247/2021).

### 2.2 Sequence analyses and epitope prediction

Epitope prediction for HLA-I and HLA-II was performed through the Immune Epitope Database (IEDB) and NetMHCIIpan 4.0, respectively (Vita et al., 2019; Reynisson et al., 2020). The most common HLA alleles in the population (coverage of > 97% for HLA-I and > 99% for HLA-II) and their respective thresholds were determined accordingly to previous studies (Weiskopf et al., 2013; Reardon et al., 2021). Epitopes with the best five scores above the threshold for each allele were annotated in the TS amino acid sequence (accession TcCLB.509495.30) and the heatmaps were generated using the libraries “os”, “re”,”seaborn”, “numpy”, “matplolib.pyplot” and “collections” for Python. The prediction of linear B cell epitopes was performed with BepiPred 3.0 (Clifford et al., 2022) and the top 20% epitopes were annotated in the TS sequence.

Sequences retrieved from TritrypDB (https://tritrypdb.org) were analyzed using the BLAST software to identify orthologues of Trans-sialidase genes in the genomes of Y, Brazil and Dm28c strains of *T. cruzi*, based on the CL Brener gene TcCLB.509495.30. Six TS sequences containing SAPA repeats (5 active and 1 inactive) were retrieved from the scaffolds generated from Illumina HiSeq paired reads and reads obtained with the PacBio platform for the CL Brener strain (Burle-Caldas et al., 2022). The prediction of domains and motifs present in the TS amino acid sequences was performed using the MEME suite tool (Bailey et al., 2009) and the number and pattern of SAPA repeats were identified using the Tandem Repeats finder software (Benson, 1999). The Figure was generated using the IBS 2.0 (Illustrator for Biological Sequences) program (Xie et al., 2022).

### 2.3 Plasmid constructions

The trans-sialidase gene sequence from the CL Brener strain of *Trypanosoma cruzi* (ID number TcCLB.509495.30) was used as a template to amplify the complete TS gene (Full-length TS) only the catalytic portion (TS without SAPA) or the only SAPA repeats of the trans-sialidase (TS-SAPA) using primers containing sites for the restriction enzymes XhoI and NheI to facilitate cloning. PCR amplification with the primers 5’-GCTAGCATGCTCTGCCCCAGCGAGCCC-3’ and 5’-CTCGAGGGGCAAAATCAAAACCGTAC-3’ was used to obtain the full-length TS sequence. The TS without SAPA was amplified using primers 5’-GCTAGCATGCTCTGCCCCAGCGAGCCC-3’ and 5’-CTCGAGCATGTGTGCTTCCGTGCC-3’, while the repeat domain (TS-SAPA) was amplified with primers 5’-GCTAGCATGGACAGCAGCAGCGACAG-3’ and 5’-CTCGAGGGGCAAAATCAAAACCGTAC-3’. All PCR products were initially cloned into TOPO-TA vector (Invitrogen), according to the manufacturer’s recommendations and subsequently sub-cloned into sites of the restriction enzymes Xhol and Nhel (Promega) in the pET21a expression vector (Novagen).

### 2.4 Expression in *E. coli* and purification of recombinant TS

*E. coli* strains (SHuffle^®^ T7 Competent) were used to express the full-length TS, TS without SAPA and TS-SAPA proteins. A single colony pre-inoculum for each bacterium was grown in 2XYT medium for approximately 18h at 37° under agitation at 180rpm, followed by a 1:100 dilution in 2XYT medium. These were grown under the same conditions until reaching an OD600 between 0.4-0.6. 0.5mM IPTG was then added and the cultures were kept inducing for about 4h. After confirmation of the induction of the recombinant proteins by SDS-PAGE, the frozen pellets corresponding to the induction of full-length TS, TS without SAPA and TS-SAPA were resuspended in Histrap binding buffer (20 mM NaH2PO4, 500 mM NaCl, 30 mM Imidazole pH 7.4), containing PMSF or protease inhibitor cocktail. These were lysed in an EmulsiFlex-C3 homogenizer (Avestin) according to the manufacturer’s instructions. The lysis product was centrifuged at 26,100 *x* g at 4°C until the lysate was clarified, obtaining the soluble (supernatant) and insoluble (pellet) fractions. The fraction containing most of the protein of interest was used for purification by affinity chromatography using a nickel column. After analyzing the fractions, these were pooled and dosed using the QUBIT 4 Fluorometer (Invitrogen, USA) device.

### 2.5 mRNA synthesis, cell transfections and encapsulation into LNPs

The Codon-optimized Tran-sialidase coding sequences, with or without the SAPA repeats and containing the appropriate 5’ and 3’ UTRs as well as a 110-nt poly-A tail, were synthesized by GenScript. The plasmids were linearized with XbaI or XhoI restriction enzyme and used as a template for *in vitro* transcription with the T7 MegaScript kit (thermo fisher scientific). Uridine was substituted with N1-methyl-pseudouridine (Trilink), and the 5’ cap was co-transcriptionally added using the CleanCap reagent (Trilink). After transcription, the mRNA was analyzed by electrophoresis on a 1% agarose gel under denaturing conditions. To analyze the expression of mRNAs by western blot, HeLa cells were cultivated in 6 well plates with a 70% confluence in Dulbecco’s Modified Eagle Medium (DMEM) and were transfected using Lipofectamine 3000 Transfection Reagent (Thermo Fisher Scientific) with 10 µg of full-length TS mRNA or TS without SAPA mRNA. As a positive control, 5 µg of pcDNA3.1 vector containing either the full-length TS sequence or TS without the SAPA domain were used. pcDNA3.0 (GenScript) encoding RFP was transfected as a transfection control. LNPs formulations were produced using a microfluidic device ANP, Particle Works, UK. The physicochemical characteristics and mRNA encapsulation efficiency are in accordance with those reported by Fernandes et al. (2025) (Fernandes et al., 2025).

### 2.6 Animal immunization and challenge

Female BALB/c mice (6–8 weeks old), obtained from the Central Animal Facility of the Federal University of Minas Gerais (UFMG), were used in the experiments. All animal procedures were approved by the Ethics Committee on Animal Use of UFMG (CEUA/UFMG) under protocol 247/2021. Mice were immunized following a prime-boost-boost protocol with 21-day intervals between doses. For each immunization strategy, animals were randomly divided into two groups (n = 5 per group). In the recombinant protein immunization protocol, mice received subcutaneous injections of 10 µg of each recombinant protein formulated with 18 µg of CpG B344 (DNA Alpha) and 30% (v/v) aluminum hydroxide (alum) in a final volume of 100 µL of saline solution. For the mRNA immunization protocol, mice were injected intramuscularly (i.m) in both hind limbs with 10 µg of LNP-mRNA encoding full-length TS, or LNP-mRNA encoding TS without the SAPA domain, in a total volume of 100 µL. Empty LNP was used as negative control group. Thirty days after the last immunization, one group of mice was challenged with 10^4^ blood trypomastigotes of *Trypanosoma cruzi* Y strain genetically modified to express luciferase. The other groups of animals were used for blood collection and splenocyte isolation, followed by *in vitro* stimulation with recombinant TS proteins to assess the cellular immune response.

### 2.7 Parasite culture, transfection with luciferase plasmid and bioluminescence imaging

A total of 4 x 10^8^ epimastigotes from the Y strain were transfected with 10 µg of pROCK vector containing the red-shifted luciferase gene and the G418 resistance gene using the Amaxa Nucleofector (Lonza). The parasites were maintained in weekly passages in Liver Infusion Tryptose (LIT) (Camargo, 1964) supplemented with 10% fetal bovine serum and 200 µg/mL of G418 until parasite selection. After selection, the parasites were subjected to luciferase assay (ONE-Glo™ Luciferase Assay System – Promega). To evaluate infection in mice, animals were injected with 100 mg kg−1 d-luciferin intraperitoneally (i.p.), anaesthetized using 2.5% (vol/vol) gaseous isofluorane in oxygen and placed in an IVIS Spectrum CT 2 *In Vivo* Imaging System (Revvity). Images were acquired 10min after d-luciferin administration and after imaging mice were revived and returned to cages. The images were analyzed using Living Image software to load the images as a group to standardize the scale. The amount of light emission was quantified using measurement ROIs.

### 2.8 Parasite quantification by qPCR

Hearts were collected 45 days after challenge with the Y strain. We performed DNA extraction with genomic DNA from tissue (Macharey-Nagel) according to the manufacturer’s instructions Macharey-Nagel. We used in the qPCR the SYBR Green protocol (Thermo Fisher) and the concentration of DNA were adjusted to 100ng per reaction. The parasite load was quantified by the standard curve method. The primers Cruzi 1 (5’-AST CGG CTG ATC GTT TTC GA- 3 ′), Cruzi 2 (5-′ AATTCCTCCAAGCAGCGGATA- 3’ were used for quantifying the *T. cruzi* DNA (Schijman et al., 2011). As an endogenous mouse gene, we used the beta actina forward (5’-CGA TGC CCT GAG GCT CTT T- 3 ′) and reverse (5’-TGG ATG CCA CAG GAT TCC AT-3’).

### 2.9 Histopathological analyses

Hearts from immunized animals were obtained 45 days after challenge with the Y strain, cut transversely, were first washed in phosphate-buffered saline (PBS; pH 7.2) and fixed using 4% phosphate-buffered formalin. The tissues were embedded in paraffin and stained with hematoxylin and eosin (H&E) for assessment of inflammation. Inflammation were evaluated, as previously described (Ricci et al., 2024). The degree of inflammation was evaluated in the atrial and ventricular free walls, as well as the interventricular septum. This was accomplished by randomly selecting five fields from H&E-stained sections at 20X objective magnification, allowing for the analysis of a myocardial area. The evaluation of fundamental pathological injuries, including signs of inflammation (e.g., inflammatory cell infiltration and edema), cell death (e.g. necrosis), tissue hyperplasia, and hemorrhage, was conducted in terms of both intensity and extent. For the histopathological analyses, we determined an histopathological score that is based on different parameters that were classified as (SD; 0), mild (1), moderate (2) and intense (3). The parameters used were: (i) the presence of inflammatory infiltrates in the heart tissue (0-3), (ii) the presence of amastigote nests (0-3), (iii) tissue degeneration, (iv) hemorrhage (0-3), (v) necrosis (0-3), (vi) edema (0-3) and (vii) hyperplasia. In the graphs we indicated only the inflammatory infiltrate parameter determined for each group.

**Figure 1:**
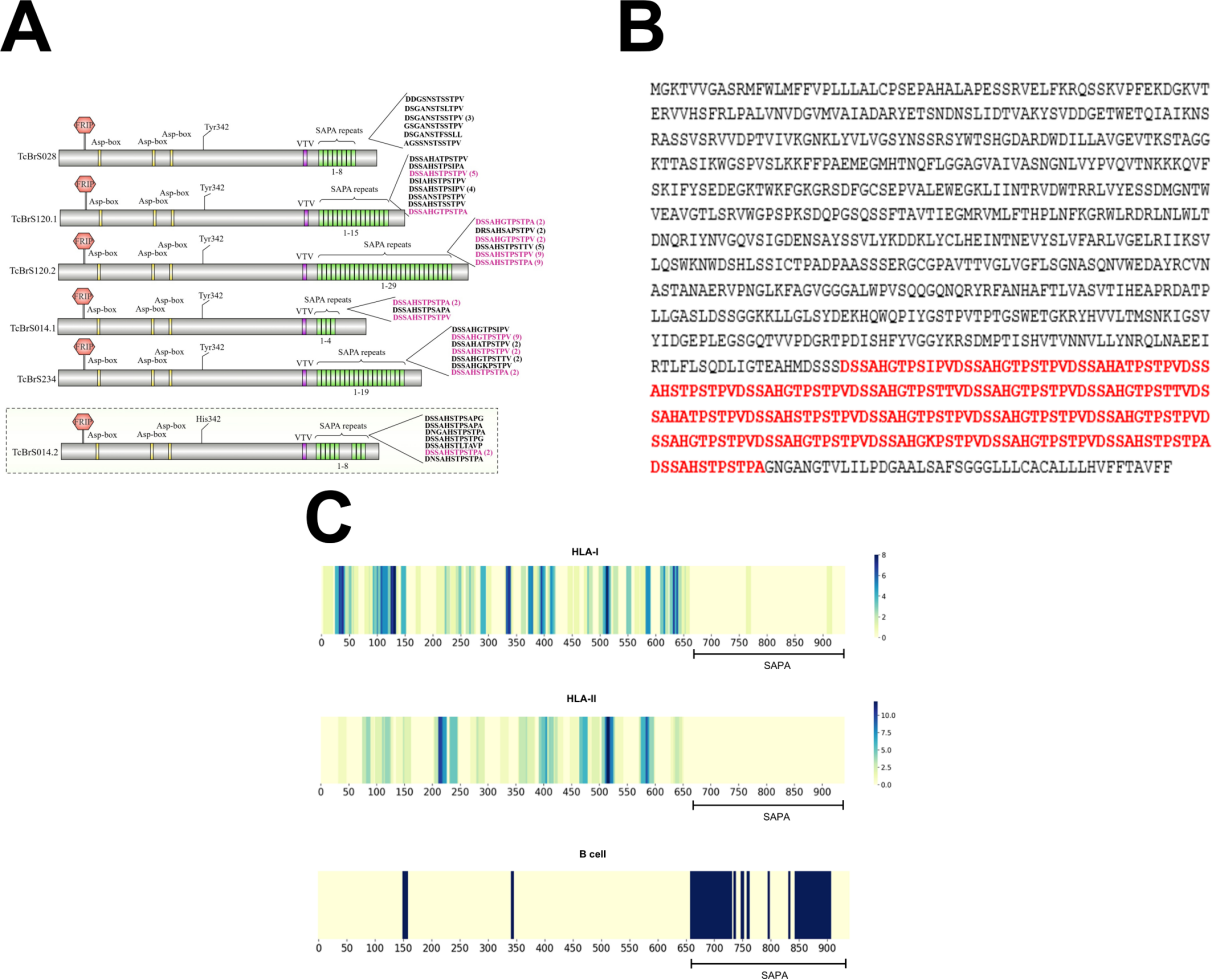
Sequence analysis of active trans-sialidases and *in silico* epitope prediction. **(A)** Analysis of SAPA repeats in the trans-sialidases from the *T. cruzi* CL Brener strain. **(B)** Amino acid sequence of an active trans-sialidase containing SAPA repeats, highlighted in red. **(C)** Predicted CD8^+^ T cell (HLA class I), CD4^+^ T cell (HLA class II), and B cell epitopes in an active trans-sialidase containing SAPA repeats, identified through *in silico* epitope prediction.

**Figure 2:**
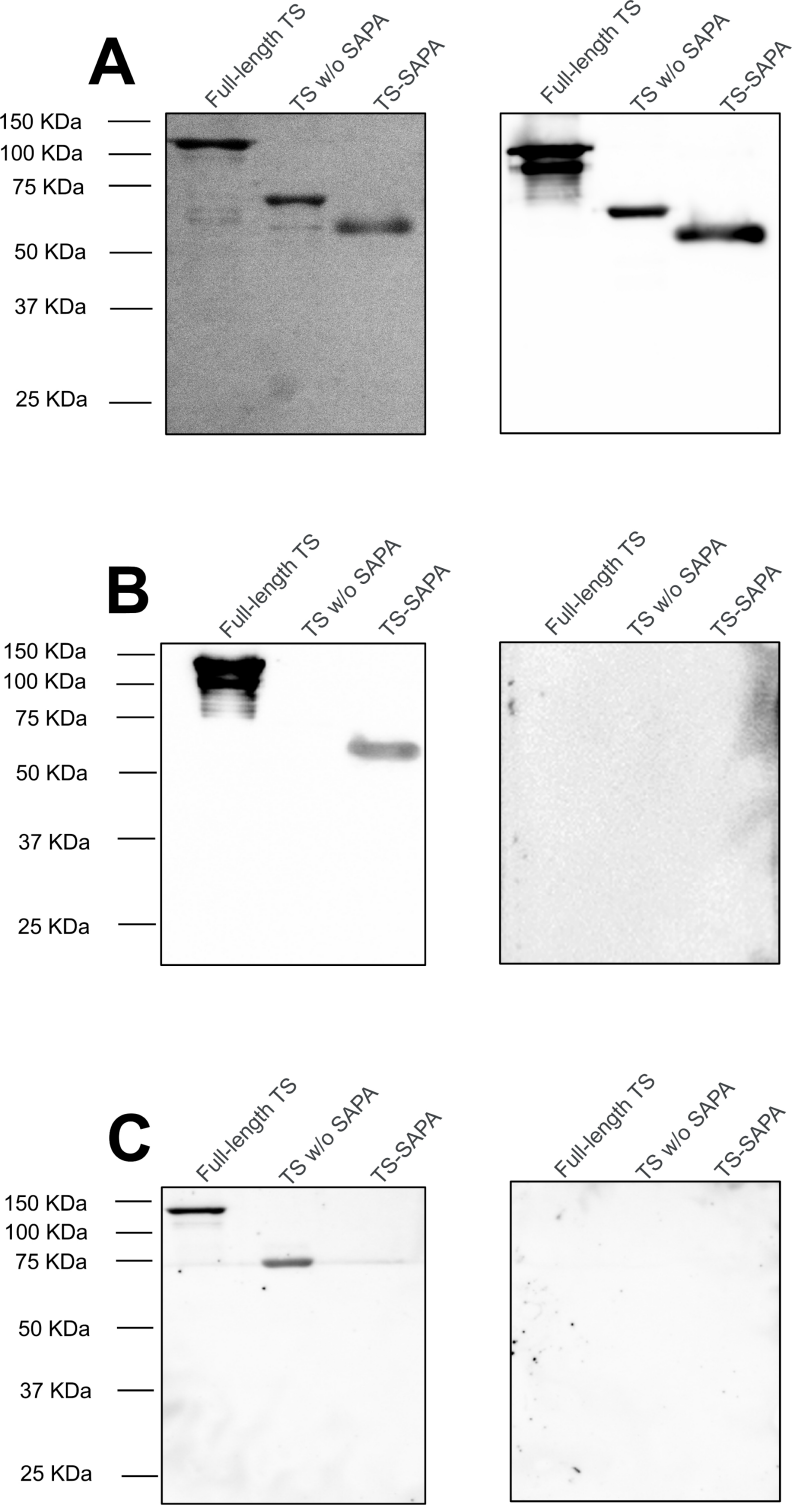
Recognition of full-length TS and truncated trans-sialidases by serum from *T. cruzi*-infected mice and patients. **(A)** SDS-PAGE showing the purification of full-length trans-sialidase, truncated TS without SAPA repeats (TS without SAPA), and only the SAPA repetitive motif (TS-SAPA) (left) and western blot using anti-His antibody (right). **(B)** Western blot of full-length and truncated TS using serum from mice in the acute phase of *T. cruzi* infection (left) and uninfected mouse serum as a control (right). **(C)** Western blot of full-length and truncated TS using serum from patients in the chronic phase of Chagas disease (left), and a negative control serum (right).

### 2.10 Western blot and ELISA

For Western blotting, proteins were separated by SDS-PAGE and transferred to nitrocellulose membranes. Membranes were blocked followed incubation primary antibodies diluted in blocking buffer. After washes with PBS-T (PBS and 0.05% Tween 20), membranes were incubated with HRP-conjugated secondary antibody (Sigma), diluted 1:5000 in blocking buffer, washed and revealed using ECL reagent (GE). For ELISA, 96-well plates were coated with 100 ng of recombinant Full-length TS, TS without SAPA or TS-SAPA proteins, reacted with sera diluted in blocking buffer followed by incubation with secondary antibodies anti-total IgG, anti-IgG1, or anti-IgG2a conjugated with streptavidin-HRP (Southern Biotech). The reactions were revealed with the substrate 3,3’ 5,5’-tetrametylbenzidine (TMB) from SIGMA and read at 450 nm.

### 2.11 Cytokine measurement

Mice spleens were collected and macerated with the aid of a metal mesh. The macerate was washed in saline and treated with ACK buffer (0.15 M NH4Cl, 0.1 M KHCO3 and 0.1 M Na2EDTA) for erythrocytes lysis. Then washed once in saline and resuspended in RPMI medium 1640 (Gibco), supplemented (complete RPMI medium) with 10% FBS (Gibco), 2 mM glutamine, 100 µg/ml streptomycin and 100 U/ml penicillin. The viability of the cells was evaluated by using 0.2% trypan blue to discriminate between live and dead cells and after counting, 1 × 10^6^ splenocytes were plated per well. These cells were then incubated with RPMI medium or concanavalin A (5 µg/mL) and restimulated with 5 µg per well with the respective proteins. After 72h, the supernatant was collected and IFN-γ and IL-10 cytokines were measured. This assay was performed by ELISA using R&D Systems Kits by enzyme linked immunosorbent assays (ELISAs), following manufacturer’s instructions.

### 2.12 Statistical analysis

Statistical analyses were performed using GraphPad Prism 8.0.1 (GraphPad Software, San Diego, California, USA). Data are shown as mean ± standard error of the mean (SEM). Data that presented normal distributions (*P* > 0.05 by Shapiro–Wilk tests) – except for parasite DNA, which was submitted to an exponential transformation and analyzed as normally distributed data – were evaluated by Student’s *t*-test, one-way and two-way analysis of variance (ANOVA), depending on the experimental design. Following significant ANOVAs, the *post-hoc* analyses were selected based on the coefficient of variation (CV): Tukey (CV ≤ 15%), Student-Newman-Keuls (CV 15–30%), and Duncan (CV > 30%). The differences were considered significant when *P* < 0.05. If distribution was not normal, t-test or Kruskal Wallis test was applied.

## 3 Results

### 3.1 Gene repertoire encoding TS in different *T. cruzi* strains and the presence of SAPA repeats

**Figure 3:**
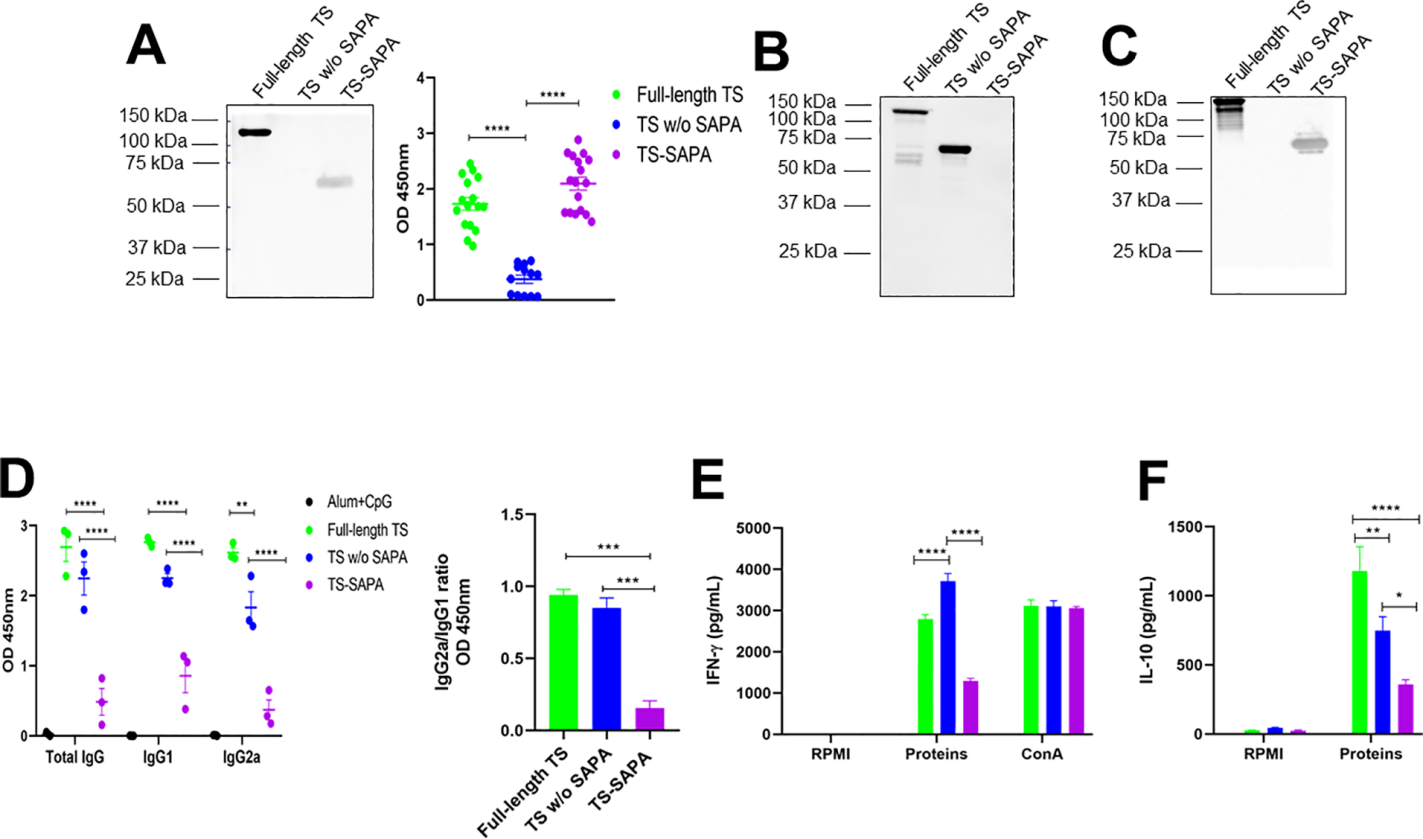
Humoral and cellular immune responses in mice immunized with different versions of recombinant TS. BALB/c mice were immunized with 10 µg of recombinant TS proteins formulated with alum and CpG adjuvants, using a prime-boost-boost protocol. Thirty days after the last immunization, sera were collected for western blot and for quantification of total IgG, IgG1, and IgG2a levels by ELISA. Spleens were also harvested for splenocyte culture and cytokine quantification. **(A)** Western blot (left) and ELISA (right) using serum from mice immunized with full-length TS against the different recombinant TS, demonstrating preferential antibody recognition of SAPA repeats over the catalytic domain. ****P < 0.0001. **(B, C)** Western blot using serum from mice immunized with TS without SAPA **(B)** and TS-SAPA **(C)**, tested against the different recombinant TS. **(D)** ELISA showing total IgG, IgG1, and IgG2a levels in serum from mice immunized with recombinant protein coated on the surface of a 96-well plate (left) and the IgG2a/IgG1 ratio (right). **P < 0.01, ***P < 0.001, ****P < 0.0001. **(E, F)** Quantification of IFN-γ **(E)** and IL-10 **(F)** in supernatants of splenocyte cultures incubated for 72h with RPMI medium (negative control), stimulated with the respective recombinant TS, or concanavalin A (ConA, positive control). *P < 0.05, **P < 0.01, ****P < 0.0001.

The identification of the complete gene repertoire of Trans-sialidases in the *T. cruzi* genome has been a challenging task due to the difficulties in assembling repetitive regions in the parasite genome. To further complicated matters, the *T. cruzi* population is highly heterogeneous, composed of a pool of strains that were classified into 6 groups also known as Discrete Typing Unit, or DTUs (Zingales et al., 2009). Since 2005, the genomes of more than 30 *T. cruzi* strains belonging to different groups have been published. In most studies, however, the final assembly did not incorporate all sequences belonging to large multigene families such as Trans-sialidases, because of gene collapsing during the assembly step. In the initial assembly of the first *T. cruzi* reference genome, from the CL Brener strain, it was estimated that this parasite contains 1430 copies of TS genes, several of them corresponding to pseudogenes (El-Sayed et al., 2005). Because the CL Brener strain is a hybrid of two distinct *T. cruzi* lineages, this number corresponds to sequences from two different alleles of each gene. Based on this dataset, we have previously analyzed 508 complete TS genes (non-pseudogenes) present in the *T. cruzi* CL Brener genome and identified eight TS groups according to the presence of key motifs including the catalytic site. These analyses revealed that only sequences belonging to TS group I, contain an intact catalytic site and thus, encode active enzymes (Freitas et al., 2011). The number of TS genes varies greatly among *T. cruzi* lineages. Recent genome analyses of other *T. cruzi* strains with improved assemblies based on long-read sequencing have shown that the genomes of Brazil clone A4 (DTU-I), Dm28c (DTU-I), Y clone C6 (DTU-II) and CL Brener (DTU-VI) have respectively 1644, 1491, 1465 and 3209 copies of TS genes (Berná et al., 2018; Wang e al., 2021). To estimate the numbers of genes encoding active TS among these different *T. cruzi* strains and DTUs, we searched for the consensus sequence encoding the TS catalytic site, which includes the conserved Tyr342 (ENSA**Y**SSVLYKDDK), located on the N-terminal of the enzyme. As shown in **Table 1**, the number of TS sequences encoding active TS enzymes varies from 2 to 14 sequences. This analysis also revealed the presence of the C-terminal SAPA repeat domain, with variable numbers of the 12 amino acid repeats, DSSAHS/GTPSTPV/A, in various TS sequences. Whereas 3 members of group I TS from the Brazil strain encode a SAPA domain with only 2 repeats in each, 5 members of group I TS present in the genome of the Dm28c strain encode TS with repeat domain containing 4, 10, 15 and 24 repeats, in which two members present four tandem copies of the SAPA motif. The Y strain has two genes encoding active enzymes but only one sequence contains a repeat domain with 29 repeats. One inactive TS containing a large repeat domain with 65 repeats was also identified in the Y strain. Among 14 predicted sequences encoding active TS present in the CL Brener genome, 5 copies have a C-terminal repeat domain with 4, 8, 15, 19 and 29 repeats, as represented in **Figure 3A**. Similar to Y strain, one copy in the CL Brener genome encodes an inactive TS containing 8 repeats. **Figure 3B** shows the amino acid sequence of one active TS isoform from CL Brener containing 19 SAPA repeats (in red), which was the sequence chosen for the immunization experiments described here. Prediction of human leukocyte antigen class-I (HLA-I) and -II (HLA-II) restricted epitopes as well as B-cell epitopes showed, as previously described, that the catalytic domain is highly enriched with T-cell epitopes whereas the C-terminal SAPA domain contains mostly B-cell epitopes (**Figure 3C**, **Supplementary Figure S1**).

**Table 1:** TS sequences in different *T. cruzi* strains.

Strain (DTU’s)	Number of TS copies	Number of active TS*	Number of active TS containing repeats	Number of inactive TS containing repeats
Brazil A4 (I)	1644	7	3 (2,2,2)	0
Dm28c (I)	1491	14	5 (4, 4, 10,15,24)	0
Y C6 (II)	1465	2	1 (29)	1(65)
CL Brener (VI)	3209**	14	5 (4,8,15,19,29)	1(8)

Number of total TS gene copies, genes encoding active TS and TS containing SAPA repeats in the genomes of four *T. cruzi* strains: Brazil clone A4 (DTU-I), Dm28c (DTU-I), Y clone C6 (DTU-II) and CL Brener (DTU-VI). *Pseudogenes were excluded. **For sequences present in the CL Brener genome, the total number of TS copies included the esmeraldo and non-esmeraldo haplotypes. Numbers in parenthesis represent the number of SAPA repeat in each TS sequence.

### 3.2 Antibody responses against the different trans-sialidases domains confirmed the immunodominance of SAPA repeats during acute infection

**Figure 4:**
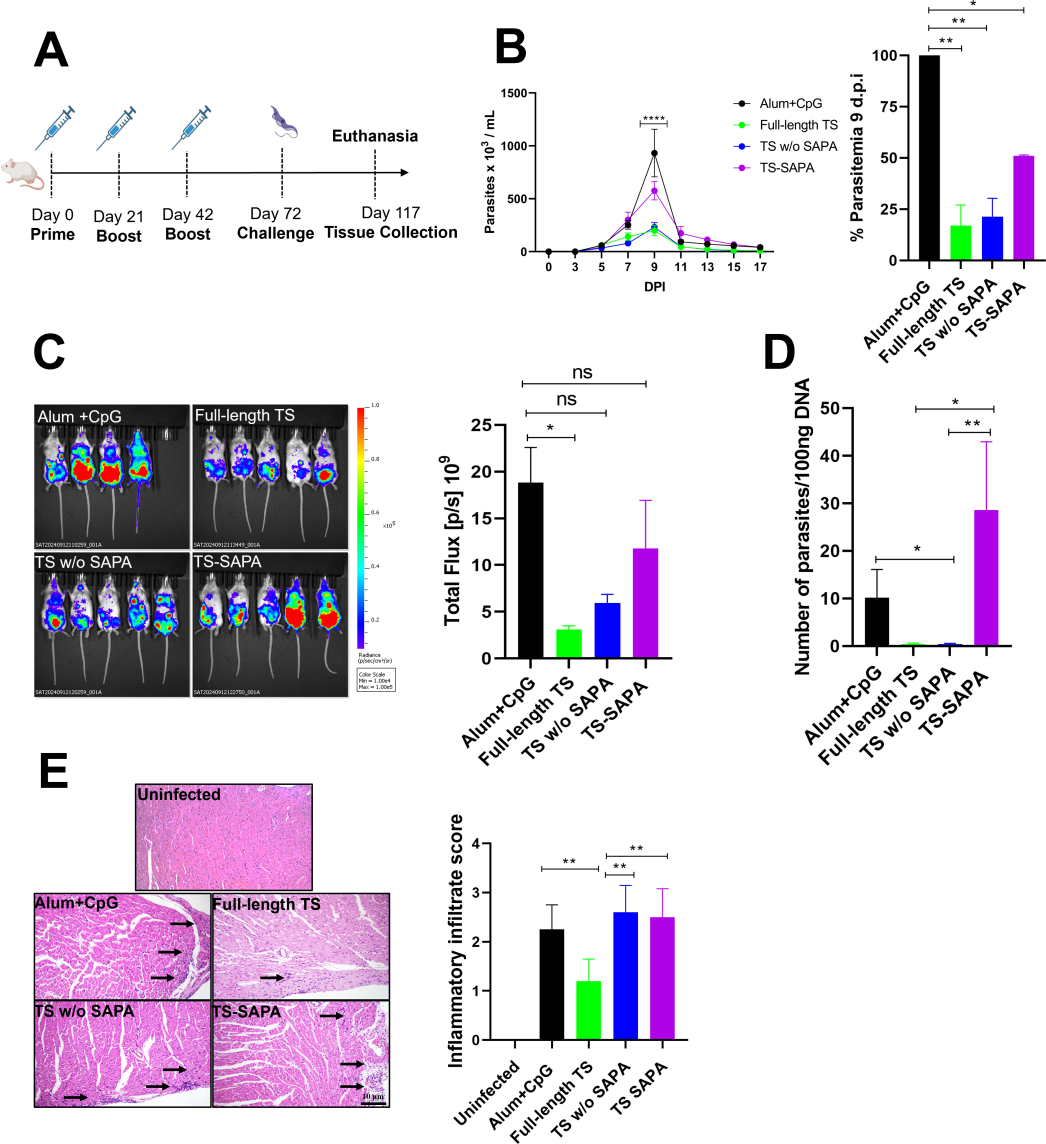
Evaluation of protection in mice immunized with TS proteins after challenge with the *T. cruzi* Y strain. **(A)** BALB/c mice were immunized using a prime-boost-boost protocol with 10 µg of recombinant TS proteins formulated with alum and CpG adjuvants. Thirty days after the last immunization, mice were challenged with 10^4^ blood trypomastigotes of *T. cruzi* Y strain expressing luciferase. **(B)** Parasitemia in immunized and challenged mice was followed for 17 days (left). A peak of parasitemia was reached at 9 days post-infection (DPI) and presented as a bar graph (right). *P < 0.05, **P < 0.01, ****P < 0.0001. **(C, D)** Tissue parasitism was assessed by bioluminescence imaging using an *In Vivo* Imaging System (IVIS) to visualize luciferase activity **(C)** and qPCR in heart tissue **(D)** from *T. cruzi* in mice that were immunized and challenged. *P < 0.05, **P < 0.01. **(E)** Histological analysis of heart tissue shown inflammatory infiltrates (arrows); inflammatory scores are shown in the bar graph (right). **P < 0.01. Scale bar = 10 μm. 20X objective.

To assess the immune response elicited by the different domains present in the TS containing SAPA repeats, we generated three versions of the recombinant protein: the protein with the N-terminal domain that contains the catalytic site and the C-terminal domain with 19 SAPA repeats (full-length TS), the protein containing the catalytic domain but lacking the SAPA repeats (TS without SAPA) and the protein containing only the C-terminal repetitive domain comprised of 19 SAPA repeats (TS-SAPA). As shown in **Figure 4A**, all recombinant proteins were expressed in *Escherichia coli* with a his-tag and purified on a Ni-column (left gel). Anti-his antibodies recognized all three recombinant proteins, full-length TS, TS without SAPA and TS-SAPA, with molecular weights of 98, 72 and 25 kDa, respectively (right gel). Mass spectrometry analyses showed that, although the TS-SAPA has a predicted MW of 25 kDa, it migrates on SDS-PAGE with apparent MW of a dimer protein with approximately 50 kDa, most likely due to the presence of the large repeat domain that is known to favor protein aggregation. Using sera from BALB/c mice infected with the Y strain, as well as sera from individuals with chronic Chagas disease, we evaluated the humoral response against the three versions of TS. As shown in **Figure 4B** (left), sera from acutely infected mice contain antibodies that recognized only the repeat domain, and do not recognize the N-terminal catalytic domain of TS. As expected, sera from uninfected mice do not recognize any of the proteins (right). In contrast, sera from chronically infected CD patients contain antibodies that recognized the N-terminal catalytic domain but not recognize the repetitive domain (**Figure 4C**, left). Sera from healthy donors do not recognize any of the proteins (right). These results confirmed the immunodominance of the SAPA domain, which occurs during the acute phase of the infection and also indicated, a delayed immune response against the catalytic domain occurs that during the chronic phase.

### 3.3 Immunization with three versions of TS reveals the capacity of the SAPA domain to direct the immune response towards an anti-inflammatory profile

**Figure 5:**
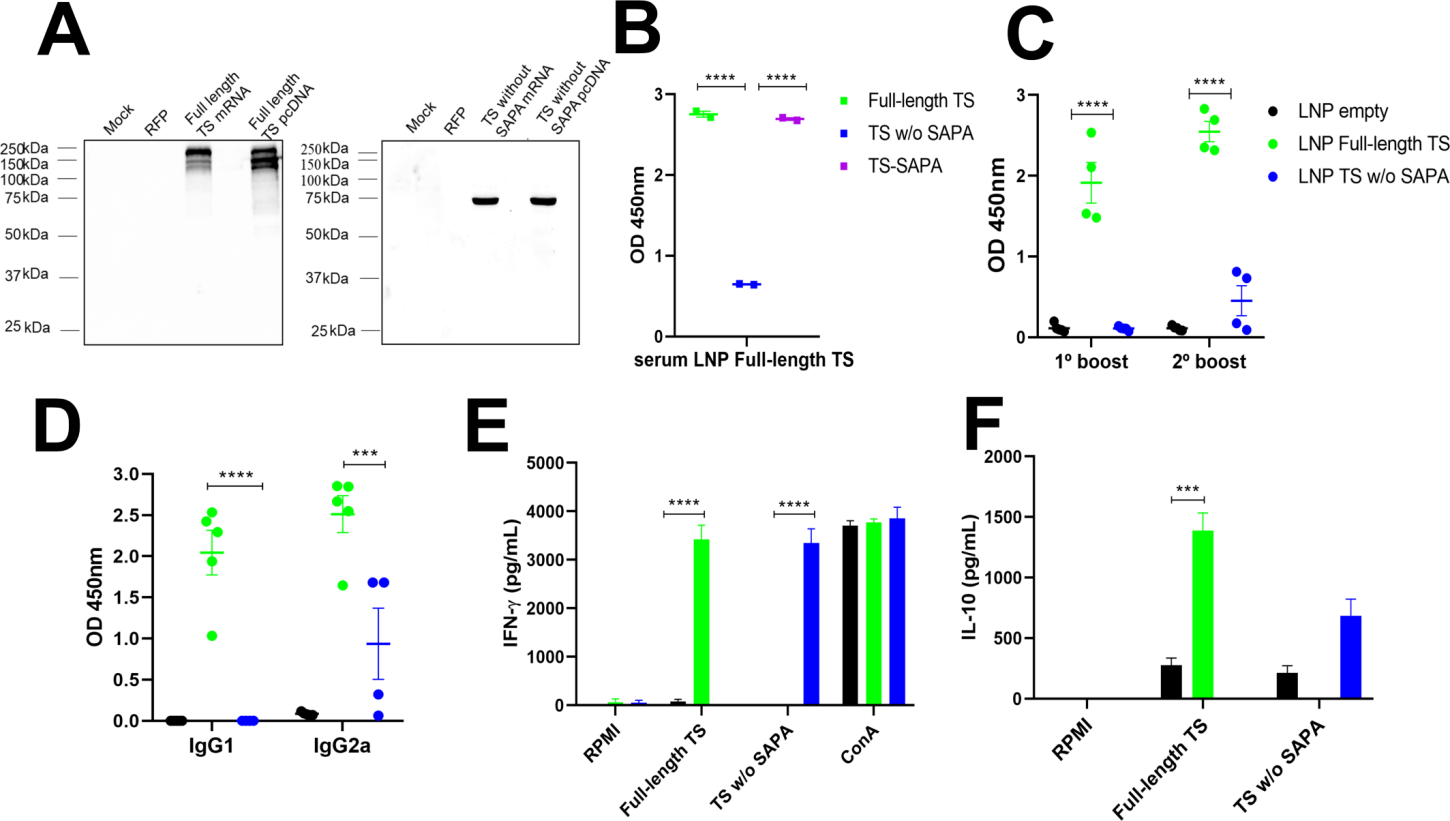
Humoral and cellular responses in mice immunized with RNA encoding TS with or without SAPA. RNA corresponding to the sequences of full-length TS and TS without SAPA repeats was transcribed *in vitro* and used in the immunization protocol. Empty LNPs were used as a control. **(A)** To assess RNA expression, HeLa cells were transfected using lipofectamine and protein expression was confirmed by Western blot using antisera from mice immunized with full-length TS (left) or TS without SAPA (right). Negative controls (Mock and Red Fluorescent Protein - RFP) and a positive control (pcDNA encoding both proteins) are shown in the Western blot. After confirming expression, RNAs were encapsulated into LNPs for use in immunization protocol. Female BALB/c mice were immunized with 10 µg of LNP-formulated RNA following a prime-boost-boost protocol as previously described. **(B)** Immunodominance of SAPA repeats was also observed with RNA formulations. ELISA was performed with serum from mice immunized with LNPs containing full-length TS RNA, which was tested against all three recombinant TS versions. Similar to protein immunization, RNA formulations induced preferential antibody recognition of SAPA repeats over the catalytic domain. ****P < 0.0001. **(C)** Serum was collected after the first and second boosts, and total IgG levels were measured by ELISA using the corresponding recombinant protein for plate coating. ****P < 0.0001. **(D)** IgG1 and IgG2a subclass levels in serum from mice immunized with TS RNA formulations were determined by ELISA. ***P < 0.001, ****P < 0.0001. **(E, F)** IFN-γ **(E)** and IL-10 **(F)** levels were quantified in supernatants from splenocyte cultures incubated with RPMI medium (negative control), stimulated for 72 h with the respective recombinant TS, or concanavalin A (ConA, positive control). ***P < 0.001, ****P < 0.0001.

To evaluate the immune response elicited by each version of the recombinant TS protein, mice were immunized with three doses of 10 µg of each TS protein version formulated with adjuvants and administered at 21-day intervals. Serum samples were collected 30 days after the last immunization, and antibody levels were subsequently analyzed. Sera from mice immunized with the full-length TS protein were incubated with the full-length TS, TS without SAPA and the TS-SAPA. As shown in the western blot (**Figure 5A**, left), antibodies from these sera specifically recognized only the full-length and the TS-SAPA, indicating that the antibody response was primarily directed against the SAPA repeats. This result was corroborated by ELISA assays (**Figure 5A**, right), confirming the immunodominance of the SAPA repetitive region. Immunization with TS without SAPA domain induced total IgG antibodies that recognized both the full-length TS and the TS without SAPA, but not the SAPA repeats. This finding indicates that, in the absence of the repetitive domain, the immune system is capable of generating antibodies against the catalytic region of the protein (**Figure 5B**). Regarding the TS-SAPA protein, incubation with proteins resulted in the recognition of only the full-length TS and the SAPA repeats, as observed with serum from mice immunized with the full-length TS (**Figure 5C**). Measurements of total IgG and its subclasses was performed by ELISA using serum from mice immunized with each recombinant TS protein and the corresponding recombinant protein coated on the surface of a 96-well plate. As shown in **Figure 5D**, both the full-length TS and the TS without SAPA domain induced high levels of total IgG and IgG1 antibodies. Both proteins also were able to induce IgG2a, but immunization with the full-length TS led to higher levels of IgG2a compared to the TS without SAPA. In contrast, immunization with the SAPA repeats alone did not induce substantial levels of total IgG or IgG2a. Despite inducing an IgG1 response, the levels remained lower than those induced by the other TS versions. The lower levels of total IgG antibodies produced by the SAPA domain alone, in contrast to the robust response elicited by full-length TS, suggests that antibody production in this context may be T cell–dependent (Millar and Kahn, 2000). We also calculated the IgG2a/IgG1 ratio and observed that the TS-SAPA protein induced a Th2-polarized response. (**Figure 5D**, right). Analysis of the cellular immune response revealed that splenocytes from mice immunized with either the full-length TS or the TS without SAPA domain, upon restimulation with the corresponding antigens, produced robust levels of IFN-γ. Notably, the response was more pronounced in the group immunized with the TS without SAPA domain. In contrast, only low levels of IFN-γ were induced by SAPA (**Figure 5E**). Remarkably, analysis of cytokine production revealed that immunization with full-length TS results in increased production of IL-10 compared to immunization with TS sequence without SAPA (**Figure 5F**). Besides, measurements of other cytokines revealed an increase in IL-6, TNF-α and IL-17 and decreased in IL-10 levels in TS without SAPA compared to full-length TS (**Supplementary Figure 2**). These results suggest the anti-inflammatory profile of the response induced by the full-length TS.

### 3.4 Immunization with three versions of recombinant TS showed that the catalytic domain is required for protection against a challenge infection

Thirty days after the last immunization, the animals were challenged with 10,000 bloodstream trypomastigote forms of the Y strain, which constitutively expresses the luciferase enzyme, and protection was subsequently assessed. The immunization protocol is shown in **Figure 1A**. Parasitemia curve (**Figure 1B**) revealed that immunization with the full-length protein and the version without SAPA repeats conferred strong protection against infection, resulting in an 83% and 78% reduction in parasitemia at the peak of infection, respectively. In contrast, immunization with TS-SAPA led to only a partial reduction, with a 48.96% decrease in parasitemia at the infection peak (**Figure 1B**, right). Because the immunized animals were challenged with the Y strain expressing luciferase, we were also able to assess parasitism through bioluminescence imaging, following the injection of the luciferin substrate into the mice. As shown in **Figure 1C**, consistent with the parasitemia results, immunization with full-length TS led to a greater reduction in bioluminescence compared to the control group that received only the adjuvant. Also, reduction to undetectable levels of parasite DNA were observed in animals immunized with the full-length antigen or the antigen containing only the catalytic domain after DNA quantification by PCR in heart tissue 45 days post-infection. In contrast, no reduction in the levels of parasite DNA was observed in animals immunized with the antigen containing only SAPA repeats and challenged with Y strain parasites (**Figure 1D**). Taken together, these results indicated that the presence of T cell epitopes located in the catalytic domain of TS is essential to confer protection after immunization with the recombinant TS protein. Histopathology analyses of heart tissues collected from animals that were immunized with the three versions of TS as well as with animals that received only the adjuvant, 45 days after challenge with the Y strain showed that the presence of SAPA repeats in the full-length TS protein resulted in a significant reduction in the inflammatory infiltrate compared to tissues from mice immunized with the antigen without SAPA and the antigen containing only SAPA. As shown in **Figure 1E**, we observed a 50% reduction in the overall score obtained in tissues from mice immunized with the full-length antigen compared to the histopathology score of heart tissues from mice immunized with the two other versions or with control mice. As expected, non-immunized and non-infected animals showed no inflammatory infiltrates. These results indicate that, although full-length TS and TS without SAPA repeats confer similar protection, the presence of SAPA repeats promotes an anti-inflammatory response that may be beneficial for long term survival of infected animals.

### 3.5 RNA immunizations confirmed the role of SAPA as an antigenic sequence that promotes an anti-inflammatory response

RNA vaccines provide a promising alternative to conventional vaccines or vaccines based on recombinant proteins because they rely on antigen production by antigen presenting cells (APCs). Composed of *in vitro* transcribed RNA encapsulated into lipid nanoparticles (LNP), RNA vaccines stimulated innate immune due to the self-adjuvant properties of the RNA and the LNP, as well as presentation via MHC class I, which ultimately leads to a strong humoral and cellular immunity (Reichmuth et al., 2016; Verbeke et al., 2022; Lu et al., 2025). To compare the protection capacity of RNA vaccines encoding TS with the protection obtained with the corresponding recombinant proteins, as well as to compare the effect of the presence of SAPA repeats in the RNA sequence, we developed RNA vaccine formulations containing the full-length TS sequence and sequence of TS without SAPA.

**Figure 6:**
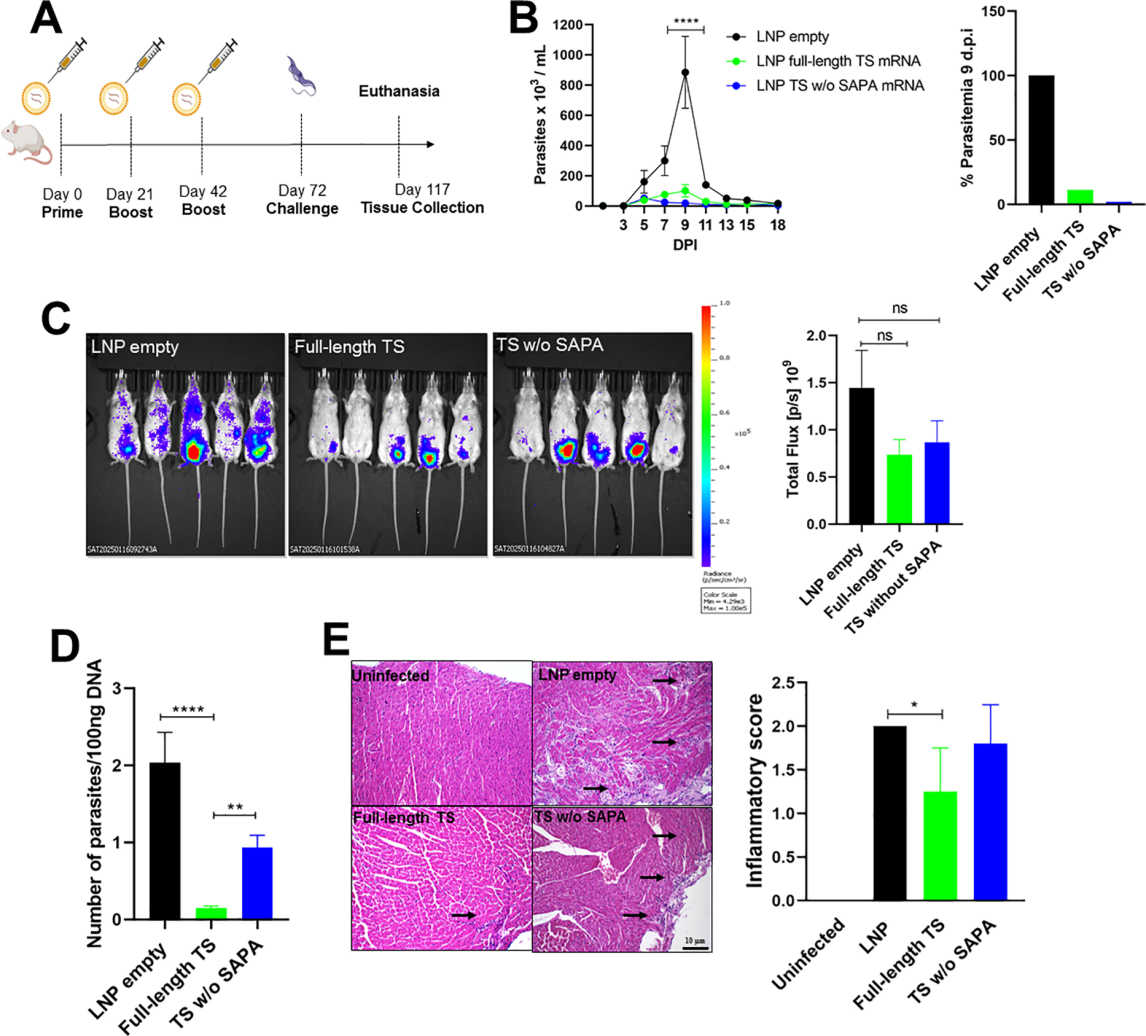
Evaluation of protection from TS RNA immunization following challenge with the *T. cruzi* Y strain. **(A)** Female BALB/c mice were immunized with LNP formulations containing 10 µg of Full-length TS RNA or TS without SAPA RNA using a prime-boost-boost protocol. Thirty days after the last immunization, mice were challenged with 10^4^ bloodstream trypomastigotes of the *T. cruzi* Y strain expressing luciferase. **(B)** Parasitemia in immunized and challenged mice was followed for 18 days (left). The peak of parasitemia was reached at 9 days post-infection (DPI) and presented as a bar graph (right). ****P < 0.0001. **(C)** Tissue parasitism was assessed by bioluminescence using an *In Vivo* Imaging System (IVIS) to visualize luciferase activity from *T. cruzi* in the mice. Bioluminescence quantification is shown on the right. **(D)** qPCR in heart tissue from immunized and challenged animals after 45 DPI. **P < 0.01, ****P < 0.0001. **(E)** Histological analysis of heart tissue showing inflammatory infiltrates (arrows); inflammatory scores are shown in the bar graph (right). *P < 0.05. Scale bar = 10 μm. 20X objective.

Before immunizing mice with RNA formulations, the *in vitro* transcribed mRNAs were tested after transfection of HeLa cells. Twenty-four hours after transfection, translation efficiencies of both mRNAs were assessed by western blots using total cell extracts of transfected cells and a polyclonal antibody obtained from mice immunized with the corresponding recombinant proteins. **Supplementary Figure S3** shows the map of the vectors containing the sequences of the full-length TS and TS without SAPA. Physicochemical properties of nanoparticles are shown in **Supplementary Table S1**. Transfection efficiency was also accessed by transfecting cells with pcDNA 3.1 plasmids containing both sequences. As shown in **Figure 6A**, high levels of expression of the full-length TS (left panel) and TS without SAPA (right panel), were observed in cells transfected with mRNA or with the pcDNAs. These results indicate that the sequences present in the *in vitro* transcribed RNA can provide efficient translation of both antigens by the cells.

Sera of animals immunized with the mRNAs encoding the full-length TS and TS without SAPA repeats formulated with LNP were used by ELISA to quantify the production of total IgG antibodies and their subclasses. As shown in **Figure 6B**, immunization with mRNA encoding the full-length TS induces the production of high IgG levels recognizing the full-length protein as well as the SAPA domain. Different from the immunization with the RNA encoding TS without SAPA, significant IgG production was detected in animals immunized with RNA encoding the full-length TS after the prime dose (not shown) and the first boost. In contrast, only low levels of antibodies were detected in animals immunized with the mRNA encoding TS without SAPA even after the 2° boost (**Figure 6C**), a result that further confirms the immunodominance of the SAPA repeats. Moreover, similar levels of IgG1 and IgG2 antibodies were detected in animals immunized with RNA encoding the full-length TS, whereas animals immunized with RNA encoding TS without SAPA produce mainly IgG2a antibodies, indicating a Th1 response profile (**Figure 6D**).

Analyses of cytokine production by splenocyte cultures from animals immunized with each RNA formulation and stimulated with the corresponding recombinant antigens also indicated that the animals immunized with RNA encoding TS without the SAPA domain display a Th1 profile, whereas animals immunized with RNA encoding the full-length TS respond with a cytokine profile that indicates an anti-inflammatory response. As shown in **Figure 6E**, splenocytes from mice immunized with RNA encoding the full-length TS or TS without SAPA that were restimulated with the respective recombinant proteins produced similar levels of IFN-γ. However, splenocytes from animals that were immunized with RNA encoding full-length TS and restimulated with the full-length protein produced higher levels of IL-10 compared to splenocytes from animals immunized with TS without SAPA RNA (**Figure 6F**). Thus, similar to the results obtained with the recombinant protein immunization protocol, RNA immunization confirms the role of SAPA repeats in the TS antigen as a sequence that promotes an anti-inflammatory response.

### 3.6 Immunization with RNA encoding full-length TS or TS without SAPA provides similar protection but results in distinct inflammatory responses

Five animals from each group that were immunized with either full-length TS RNA or TS without SAPA RNA were challenged with 10,000 trypomastigotes of the Y strain expressing luciferase (**Figure 2A**). After the challenge infection, analyses of parasitemia, followed for 18 days, showed a 90% reduction in animals immunized with full-length TS RNA, as well as in those immunized with TS without SAPA RNA (**Figure 2B**). Although it needs to be confirmed in a parallel immunization experiment, this reduction in parasitemia was more pronounced compared with the protection observed in animals immunized with the recombinant protein. Like the results obtained when animals were immunized with recombinant proteins, we did not detect any significant difference between the two groups of immunized animals, if we compare either protection levels based on the numbers of parasites in the blood or protection based on the bioluminescence signal in the infected mice at day 14 after challenge (**Figure 2C**). However, DNA quantification in heart tissues determined 45 days after the challenge showed that animals immunized with RNA encoding full-length TS have less parasite DNA compared to animals immunized with RNA encoding TS without SAPA, or with the control group, i.e., animals that received the empty LNP (**Figure 2D**).

Also similar to the results described in the immunization experiments with recombinant proteins, histopathology analyses of heart tissues collected on day 45 post-infection showed significantly lower inflammatory infiltrates in animals immunized with the RNA encoding full-length TS compared to animals that received the empty LNP (**Figure 2E**). These results are in accordance with the increased IL-10 production by animals immunized with the full-length TS RNA. Since immunization with both RNA formulations resulted in similar production of IFN-γ and similar protection regarding parasitemia, but a lower parasite DNA and inflammatory infiltrate in the heart tissue of animals immunized with RNA encoding full-length TS compared to immunization with RNA encoding TS without SAPA, it can be assumed that the presence of SAPA repeats in the RNA formulation may be beneficial when we design an RNA vaccine for CD.

## 4 Discussion

Antigens containing repeat domains have been frequently described in studies of the immune response in parasitic infections such as malaria and leishmaniasis. The *Plasmodium falciparum* circumsporozoite protein (PfCSP), which covers the surface of Plasmodium sporozoites, is one of the well characterized parasite antigens that contains tandem amino acid repeats. PfCSP has a largely disordered central region composed of five amino acids (asparagine, alanine, valine, aspartate and proline) with the immunodominant motifs NANP, NPDP and NVDP repeated 40–46 times. In addition to CSP, another 25 Plasmodium genes have been identified as genes encoding proteins with repetitive domains, in which the repeat consensus varies from 3 to 21 amino acids (Davies et al., 2017).

Several *Leishmania* proteins, including two antigens currently used in the diagnosis as well as in studies towards vaccine development, the *L. infantum* kinesin-related antigen K39, and the amastigote specific A2 protein, are also composed by large repetitive domains. K39 is composed of an immunogenic epitope with several copies of a tandemly arrayed 39 amino acid repeats (Siqueira et al., 2021). The *L. infantum* A2 protein, the antigen component of the Leish-Tec^®^ vaccine, a commercially available vaccine against canine visceral leishmaniasis, is composed predominantly of a sequence encoding 10 amino acids that are repeated 40 to 90 times depending on the *Leishmania* species (Zhang et al., 2003; Fernandes et al., 2012).

Although high antibody levels against repetitive proteins of the parasite in endemic regions do not provide protection against the disease, it is speculated that the repetitive domains in the parasite’s antigens may even be detrimental to an effective immune response by crosslinking B-cell receptors and promoting an inferior, T-cell-independent immune response. This is the basis of the antigenic smokescreen hypothesis, according to which several cross-reactive repetitive antigens impair the maturation of the affinity of antibodies against protective antigens (Kemp et al., 1987; Fernandes and Andrews, 2012; Davies et al., 2017; Siqueira et al., 2021). Despite that, many repetitive sequences that elicit strong humoral responses, particularly those located on the parasite surface, such as the *Plasmodium* CSP antigen, have been tested as vaccine components for parasitic diseases. Inhibitory antibodies against the CSP repeats, known to be protective in mouse models of infection, exhibit homotypic interactions, i.e., interactions between the variable domains of two antibodies when they are bound to adjacent repetitive epitopes (Kucharska et al., 2022). It has been shown that several copies of Fab can bind the CSP repeat simultaneously, suggesting that the extensive homotypic interactions between densely packed CSP-bound Fabs may improve affinity to the antigen and increase protection.

A search in the *T. cruzi* genome database revealed that this parasite genome is also rich in sequences encoding proteins with repetitive domains, with a total of 357 genes containing amino acid repeats (Goto et al., 2008). In contrast to *P. falciparum*, which have a prevalence of protein repeat domains with short period sizes, i.e., with the length of the repeat unit smaller than 72 bp, in the *T. cruzi* and *L. major* genomes, have a wide distribution of period sizes (Goto et al., 2008). Among the top 20 *T. cruzi* genes with a repeat domain, at least 13 of them encode antigens that are recognized by antibodies from Chagas’ disease patients. After immunoscreening a *T. cruzi* library with sera from chronic CD patients, we have previously identified several novel antigens that are target of the humoral immune response and this study also showed that 70% of them, including ribosomal, flagellar and heat shock proteins, contain repeat domains (DaRocha et al., 2002). Another study based on genome sequences (Goto et al., 2008) also revealed that 40% of the *T. cruzi* genes with repetitive domains belong to the three largest gene families encoding surface proteins named trans-sialidase (TS), mucins and MASPs. Because TS have been widely tested as a vaccine candidate for CD, we decided to investigate the impact of the presence of the SAPA domain on the protective response of vaccines based on TS recombinant proteins or TS mRNA.

The C-terminal SAPA domain with variable number of repeat units was identified in members of the TS family from different *T. cruzi* strains many years ago using southern blot analyses (Frasch, 2000). After the complete genome of the CL Brener became available, we found that most TS genes that encode active enzymes contain the C-terminal SAPA repeat domain (Freitas et al., 2011). Because this observation was obtained from sequence analysis of only one parasite strain, the CL Brener strain, we decided to verify whether it holds true for other *T cruzi* strains. Our results showed that this is also the case for another three strains, two of them belonging to the divergent *T. cruzi* lineage TcI, Brazil and Dm28c as well as for the Y strain, which belongs to TcII and is a strain widely used in studies towards vaccine development, including this one. While the numbers of TS gene copies encoding active enzymes are similar for all strains, except for the Y strain, the number of repeats within the sequences of active enzymes is greater than 2, except for the Brazil strain. It is noteworthy that Y strain has only two sequences encoding active enzymes and only one copy has SAPA repeats whereas one copy encoding an inactive enzyme contains 65 SAPA repeats. *T. cruzi* virulence varies widely among strains, but no clear correlation could be established between virulence and the presence of SAPA repeats.

A recent study from our group, based on CRISPR disruption of 14 genes from the CL Brener strain that encode active TS showed that TS activity is essential for parasite survival in the mammalian host (Burle-Caldas et al., 2022). TS mutants with undetectable levels of TS activity did not show differences in their capacity to invade cells, compared to wild type parasites, but have impaired differentiation of intracellular amastigotes into trypomastigotes. We did not observe the establishment of infection in mice WT and IFN-γ knockout immunized with TS mutants. Immunization with these TS mutants after challenge with *T. cruzi* (Y strain) also fully protected the animals. Since TS mutants also do not express TS proteins with SAPA repeats, as shown by immunostaining with anti-SAPA antibodies, it remains to be investigated whether re-expression, in the mutant, of active enzymes without SAPA repeats is sufficient to revert its attenuated phenotype. These studies are underway.

Here, the immunodominance of the SAPA repeats was confirmed since mice immunized with the full-length TS recombinant antigen or RNA encoding full-length TS produced antibodies that recognize only the protein containing the repeats and do not recognize the catalytic, non-repetitive domain of TS. Only when mice were immunized with the antigen that does not contain the SAPA domain do we observe a response against the non-repetitive domains. We also observed that animals immunized with the recombinant TS protein without SAPA exhibited higher levels of total IgG compared to those immunized with RNA. This outcome can be attributed to the fact that immunization with the recombinant protein results in antigen presentation via the MHC class II pathway, whereas RNA immunization requires intracellular translation of the antigen, and, as a consequence, is preferentially presented via the MHC class I pathway (Cunha-Neto, 1999). Although analyses of IgG subclasses in sera from mice immunized with different versions of the recombinant protein did not show significant differences, the humoral response in mice immunized with RNA formulations containing the two distinct TS sequences clearly showed a higher IgG2a/IgG1 ratio in animals that were immunized with the TS sequence that does not encode the SAPA domain. This change in the Th1/Th2 profiles is more evident when we analyzed cytokine production. Mice immunization with the protein or RNA formulation containing the SAPA domain promotes higher IL-10 and lower IFN-γ productions compared to the immunization with the protein or RNA without repeats. As expected, immunization with the recombinant antigen containing only SAPA repeats results in very low humoral responses, with high IgG1 production and low IFN-γ production since it does not contain T-cell epitopes. Also, this weak immune response after immunization with the recombinant antigen containing only SAPA repeats is not protective, in contrast to the significant protection observed in mice immunized with the full-length protein or RNA or the TS protein or RNA without SAPA. Although similar protection rates were observed in mice immunized with protein or RNA with or without SAPA, a balanced inflammatory response due to the presence of the immunodominant SAPA repeat was observed. As shown by the histopathology analyses, the increased IL-10 production in animals immunized with the full-length protein or RNA encoding full-length TS may contribute to lowering the damage caused by the intense inflammation and Th1-like response that is commonly observed in patients with chronic Chagas cardiomyopathy (Koh et al., 2023). The role of IL-10 as a protective factor in *T. cruzi* infection has been revealed by many studies in animal models of infection (Hunter et al., 1997; Costa et al., 2009; Ruiz Díaz et al., 2015). Using two distinct mouse strains as well as IL-10–deficient mice, Roffê et al. (2012) have shown that differences in IL-10 expression strongly correlate with protection from fatal acute myocarditis (Roffê et al., 2012). Our vaccination protocols that resulted in significant protection against infection induced the expression of multiple cytokines including IL-6, IFN-γ, TNF and IL-17, which have been associated with a more effective control of *T. cruzi* infection (Sanchez Alberti et al., 2020). It is noteworthy the increase in IL-17 since this cytokine activates NADPH oxidase, leading to the production of reactive oxygen species (ROS), which protect cells by eliminating intracellular parasites (Cai et al., 2016). It is also important to note that the anti-inflammatory profile observed when full-length TS sequences were used during immunization may contribute to a balance immune response, which is known to have a critical influence on shaping the clinical manifestation and determining the severity of the disease outcome (Cai et al., 2016).

Previous work from our group has also demonstrated that immunization with another *T. cruzi* antigen containing a repetitive domain resulted in increased IL-10 production and lower parasite numbers compared to the same antigen without repeats (Toro Acevedo et al., 2017). TcRpL7a is a *T. cruzi* ribosomal protein with a N-terminal Ala, Lys, Pro-rich repeat domain. Mice immunized with the full-length TcRpL7a recombinant protein were partially protected against the infection whereas immunization with TcRpL7 without repeats did not significantly alter parasitemia levels compared to controls. Although we have not analyzed the tissues from these mice, we also detected higher IL-10 production in mice immunized with the full-length TcRpL7a, i.e., with the antigen containing the repetitive domain, compared with mice immunized with the protein without the repeat domain (Toro Acevedo et al., 2017). This study strengthens the hypothesis that the presence of the repeat domain may contribute to a more balanced inflammatory response and thus should be included if we plan to design a vaccine candidate against CD. Several studies have shown that the repetitive domains of *T. cruzi* TS are highly immunogenic and capable of modulating host immunity by diverting the humoral responses to the repeat domain and exerting immunoregulatory effects (Ruiz Díaz et al., 2015; Toro Acevedo et al., 2017; Girard et al., 2021). These effects include the expansion of regulatory B and T cells and increased IL-10 production, consistent with the activity attributed to the SAPA domain. Various mechanisms responsible for the induction of IL-10 in the presence of the SAPA domain of the TS antigen could be considered, including the expansion of regulatory B and T cells. It can be hypothesized that the repeat domain induces Foxp3 expression, promoting the expansion and activation of Tregs and/or Bregs with enhanced IL-10 secretion. One possible mechanism may involve SAPA interactions with Toll like receptors such as TLR-2 on innate immune cells (e.g., dendritic cells, macrophages) or in B cells triggering IL-10 release. *In vitro* experiments with the *T. cruzi* antigen Tc52, which also contains a repeat domain, have shown that Tc52 interacts with innate immune cells via TLR-2 and modulates T cell proliferation and IL-10 mRNA expression (Ouaissi et al., 2002). This cytokine may then act on T and B cells through their cytokine receptors and preferentially drives the differentiation of anti-inflammatory subsets, including Th2 and Treg cells. Another possibility is cross-linking of B cell receptors mediated by SAPA repeats resulting in B cell activation and IL-10 production as suggested by the experiments describing TS-induced B cell activation *in vitro* and IL-10 production (Gao et al., 2002). Clearly, further studies are required to investigate the molecular mechanisms involved in shaping the inflammatory profile in response to the presence of the repeat domain in these antigens.

Different groups have tested members of the trans-sialidases family that do not contain SAPA repeats in their composition, obtaining promising results. Plasmid DNA vaccination with the gene encoding amastigote surface protein 2 (ASP-2), a member of the TS family that does not have TS activity, as well as a three-dose vaccination regimen with a recombinant fusion protein with different portions of the ASP-2 sequence containing T cells epitopes result in similar protection (up to 100% survival) against the lethal *T. cruzi* infection in the highly susceptible A/Sn mice (Araújo et al., 2005). Other authors have tested prophylactic as well as therapeutic vaccines based on replication-defective human Type 5 recombinant adenoviruses (rAd) carrying sequences of ASP-2 and TS that resulted in protection against a challenge with the Y and Colombia *T. cruzi* strains and reversion of cardiomyopathy due to reduced numbers of perforin+ T cells in the heart (Pereira et al., 2015). Intranasal vaccination with recombinant TS associated with CpG was also able to increase IFN-γ production in immunized animals and resulted in 100% protection against challenge (Hoft et al., 2007). Recently, we demonstrated that a chimeric protein, named TRASP, which contains the most immunogenic regions for T and B cells from TS and ASP-2 and includes three SAPA repeats, induces high levels of *T. cruzi*-specific antibodies and IFN-γ–producing primarily by CD8^+^ T cells and protection of the animals. The TRASP formulation showed not only strong protection in mice against various *T. cruzi* strains that lasted for at least 3 months after immunization but is also protective in dog models of CD (Castro et al., 2023). The study presented here, suggesting the involvement of the SAPA repeat domain in promoting a balanced inflammatory response contributes to establish the best formulations to be tested in a CD vaccine. Finally, our work also validates the use of RNA formulations as a novel and highly efficient alternative for the development of this much needed vaccine.

## Data Availability

The protein sequence used in the article has been deposited in the TriTrypDB database under the ID TcCLB.509495.30.
