# Correction: RNA and protein immunization with *Trypanosoma cruzi* trans-sialidase containing SAPA repeats protects mice against infection and promotes a balanced inflammatory response

**DOI:** 10.3389/fcimb.2025.1729034

**Published:** 2025-11-06

**Authors:** Nailma Silva Aprigio dos Santos, Carlos Roberto de Almeida-Júnior, Mayra Fernanda Ricci, Rodrigo C. O. Sanches, Renata Salgado Fernandes, Gabriela de A. Burle-Caldas, Júlia Teixeira de Castro, João Luís Reis-Cunha, Daniella C. Bartholomeu, Ana Clara Martins Meira, Thaiane Gomes Nascimento, Natalia Fernanda de Melo Oliveira, Ricardo T. Gazzinelli, Fabiana S. Machado, Santuza M. R. Teixeira

**Affiliations:** 1 Departamento de Bioquímica e Imunologia, Universidade Federal de Minas Gerais, Belo Horizonte, Brazil; 2 Centro de Tecnologia de Vacinas, Universidade Federal de Minas Gerais, Belo Horizonte, Brazil; 3 Department of Biology and York Biomedical Research Institute, University of York, York, United Kingdom; 4 Departamento de Parasitologia, Universidade Federal de Minas Gerais, Belo Horizonte, Brazil

**Keywords:** Chagas disease, trans-sialidase, SAPA repeats, RNA, LNP, vaccine

**Figures 1**, **2**, **3**, **4**, **5** and **6** were in the wrong order. **Figure 1** and its respective caption correspond to **Figure 4**. **Figure 2** and its respective caption correspond to **Figure 6**. **Figure 3** and its respective caption correspond to **Figure 1**. **Figure 4** and its respective caption correspond to **Figure 2**. **Figure 5** and its respective caption correspond to **Figure 3**. **Figure 6** and its respective caption correspond to **Figure 5**. The order has now been corrected.

**Figure 1:**
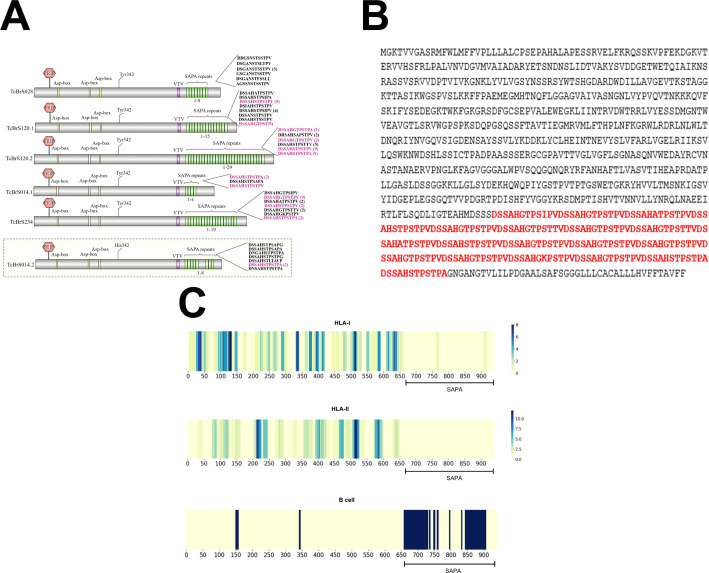
Sequence analysis of active trans-sialidases and *in silico* epitope prediction. **(A)** Analysis of SAPA repeats in the trans-sialidases from the *T. cruzi* CL Brener strain. **(B)** Amino acid sequence of an active trans-sialidase containing SAPA repeats, highlighted in red. **(C)** Predicted CD8^+^ T cell (HLA class I), CD4^+^ T cell (HLA class II), and B cell epitopes in an active trans-sialidase containing SAPA repeats, identified through *in silico* epitope prediction.

**Figure 2:**
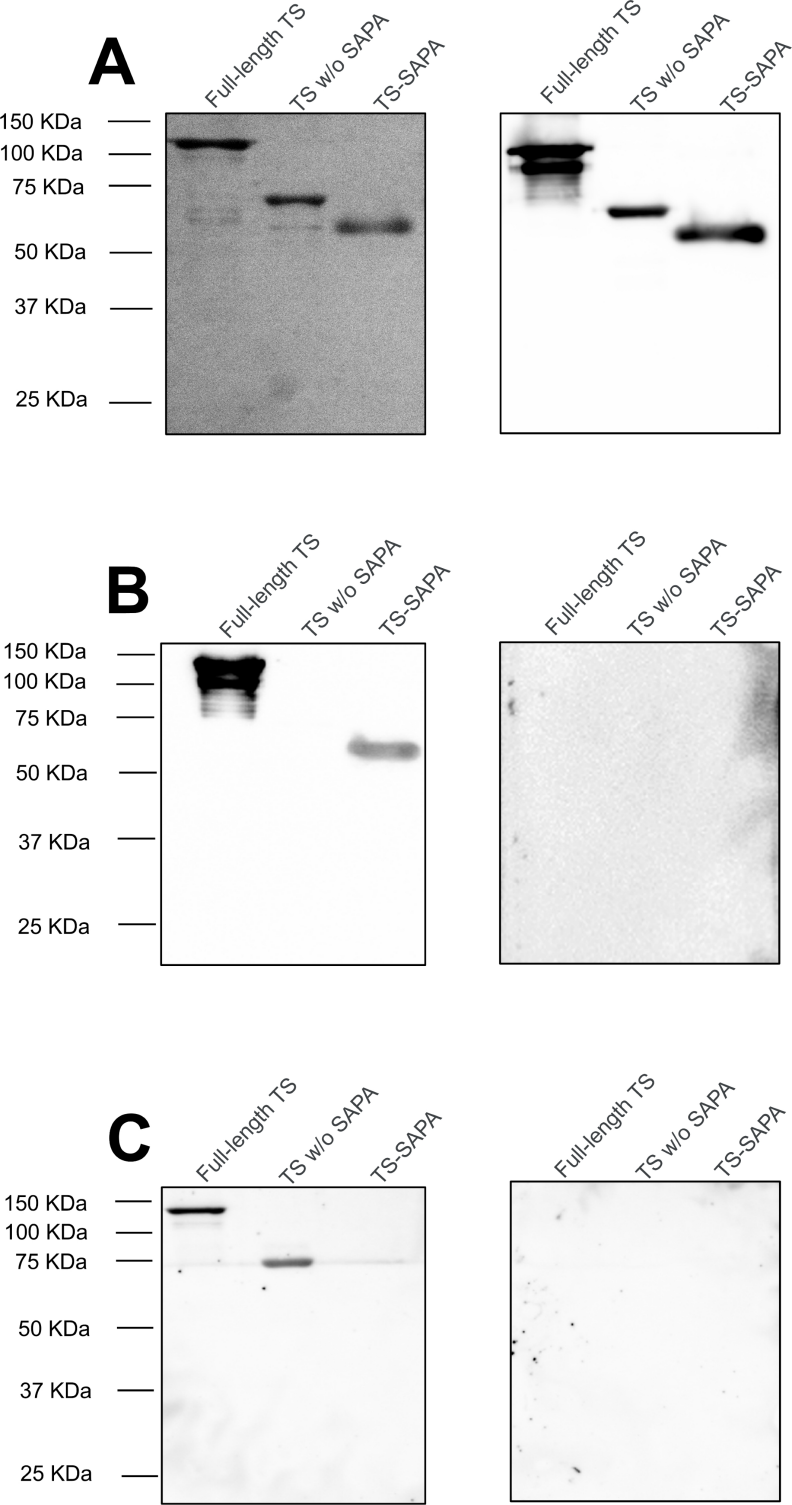
Recognition of full-length TS and truncated trans-sialidases by serum from *T. cruzi*-infected mice and patients. **(A)** SDS-PAGE showing the purification of full-length trans-sialidase, truncated TS without SAPA repeats (TS without SAPA), and only the SAPA repetitive motif (TS-SAPA) (left) and western blot using anti-His antibody (right). **(B)** Western blot of full-length and truncated TS using serum from mice in the acute phase of *T. cruzi* infection (left) and uninfected mouse serum as a control (right). **(C)** Western blot of full-length and truncated TS using serum from patients in the chronic phase of Chagas disease (left), and a negative control serum (right).

**Figure 3:**
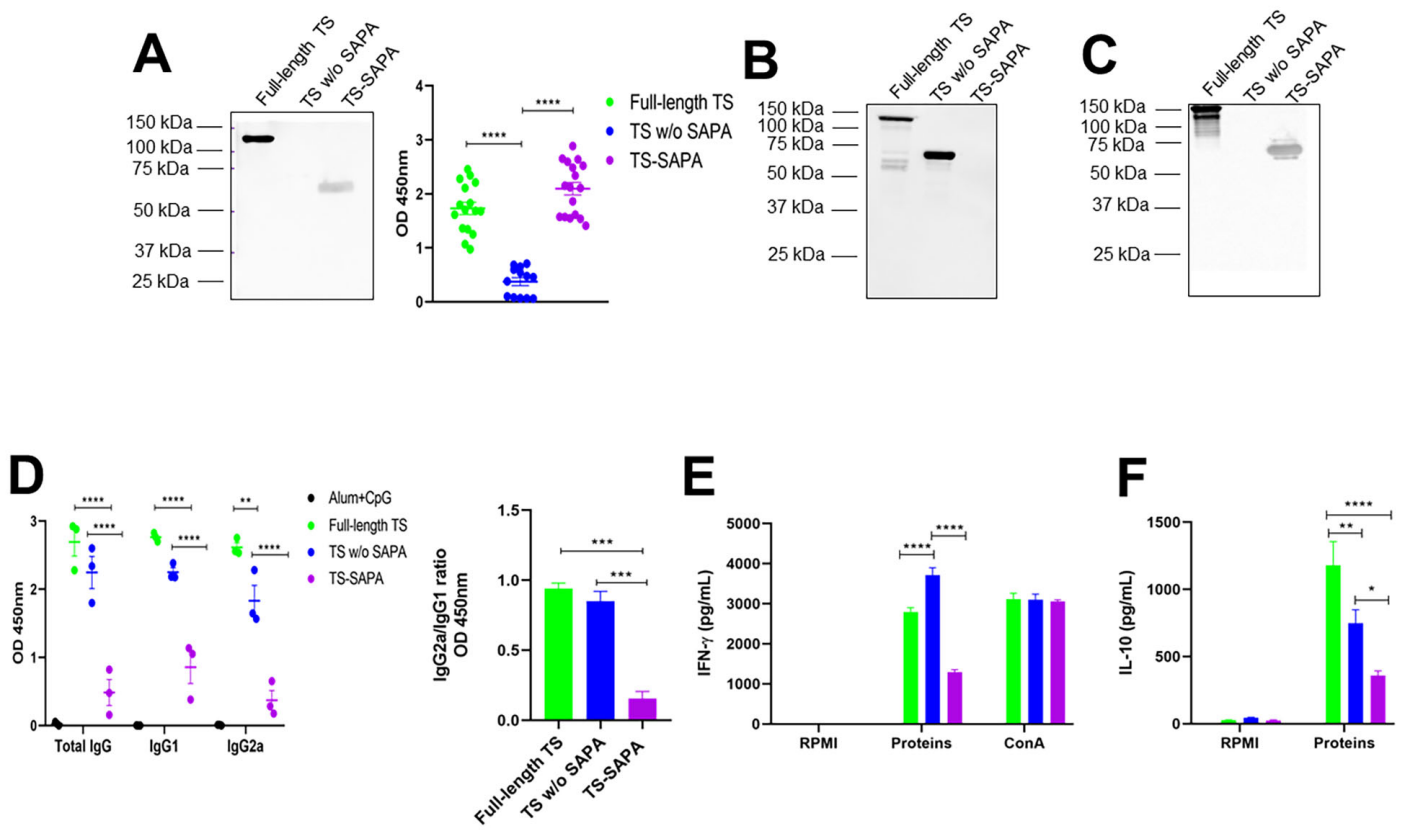
Humoral and cellular immune responses in mice immunized with different versions of recombinant TS. BALB/c mice were immunized with 10 μg of recombinant TS proteins formulated with alum and CpG adjuvants, using a prime-boost-boost protocol. Thirty days after the last immunization, sera were collected for western blot and for quantification of total IgG, IgG1, and IgG2a levels by ELISA. Spleens were also harvested for splenocyte culture and cytokine quantification. **(A)** Western blot (left) and ELISA (right) using serum from mice immunized with full-length TS against the different recombinant TS, demonstrating preferential antibody recognition of SAPA repeats over the catalytic domain. ****P < 0.0001 **(B, C)** Western blot using serum from mice immunized with TS without SAPA **(B)** and TS-SAPA **(C)**, tested against the different recombinant TS. **(D)** ELISA showing total IgG, IgG1, and IgG2a levels in serum from mice immunized with recombinant protein coated on the surface of a 96-well plate (left) and the IgG2a/IgG1 ratio (right). **P < 0.01, ***P < 0.001, ****P < 0.0001. **(E, F)** Quantification of IFN-γ **(E)** and IL-10 **(F)** in supernatants of splenocyte cultures incubated for 72h with RPMI medium (negative control), stimulated with the respective recombinant TS, or concanavalin A (ConA, positive control). *P < 0.05, **P < 0.01, ****P < 0.0001.

**Figure 4:**
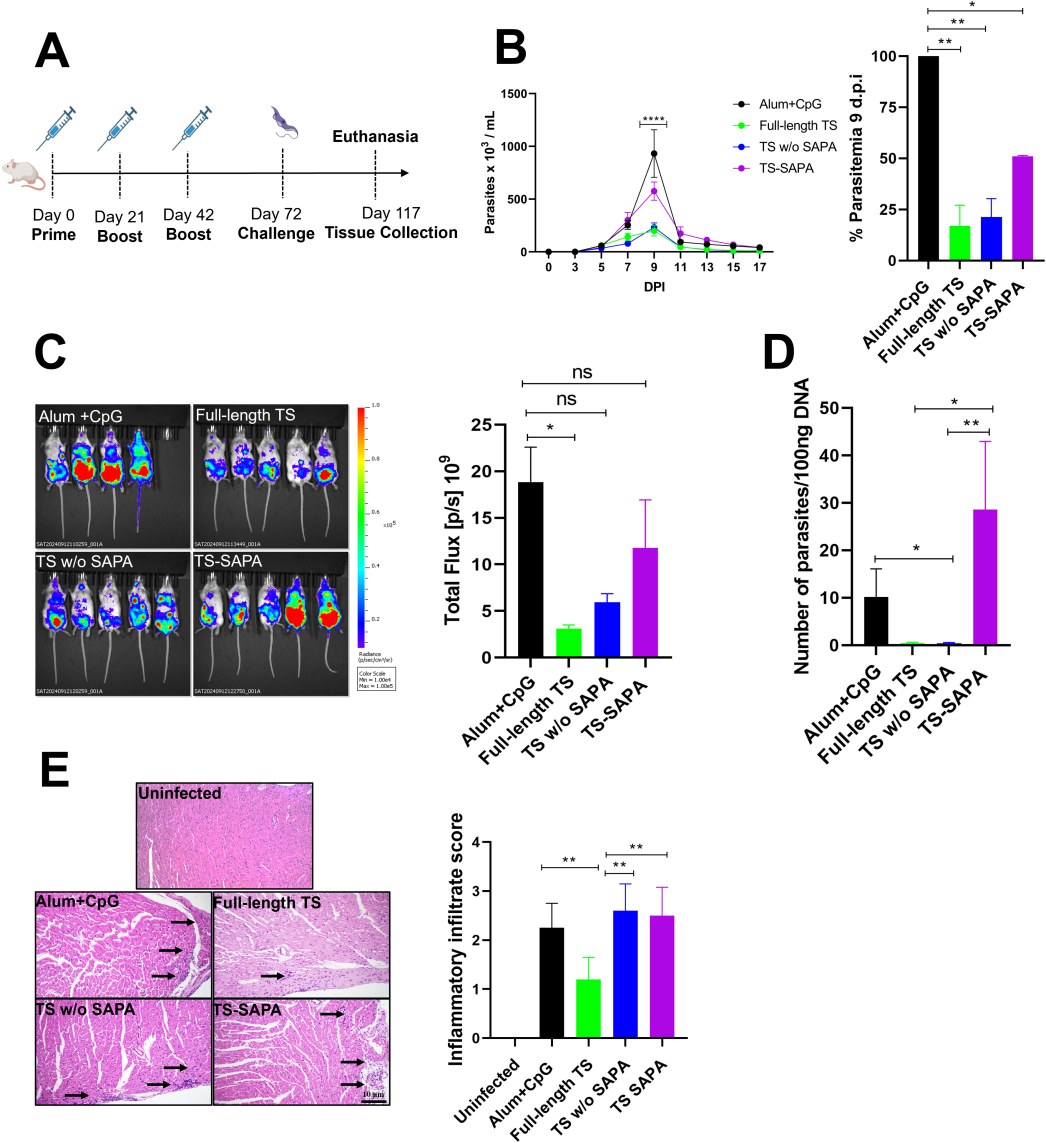
Evaluation of protection in mice immunized with TS proteins after challenge with the *T. cruzi* Y strain. **(A)** BALB/c mice were immunized using a prime-boost-boost protocol with 10 μg of recombinant TS proteins formulated with alum and CpG adjuvants. Thirty days after the last immunization, mice were challenged with 10^4^ blood trypomastigotes of *T. cruzi* Y strain expressing luciferase. **(B)** Parasitemia in immunized and challenged mice was followed for 17 days (left). A peak of parasitemia was reached at 9 days post-infection (DPI) and presented as a bar graph (right). *P < 0.05, **P < 0.01, ****P < 0.0001. **(C, D)** Tissue parasitism was assessed by bioluminescence imaging using an *In Vivo* Imaging System (IVIS) to visualize luciferase activity **(C)** and qPCR in heart tissue **(D)** from *T. cruzi* in mice that were immunized and challenged. *P < 0.05, **P < 0.01. **(E)** Histological analysis of heart tissue shown inflammatory infiltrates (arrows); inflammatory scores are shown in the bar graph (right). **P < 0.01. Scale bar = 10 μm. 20X objective.

**Figure 5:**
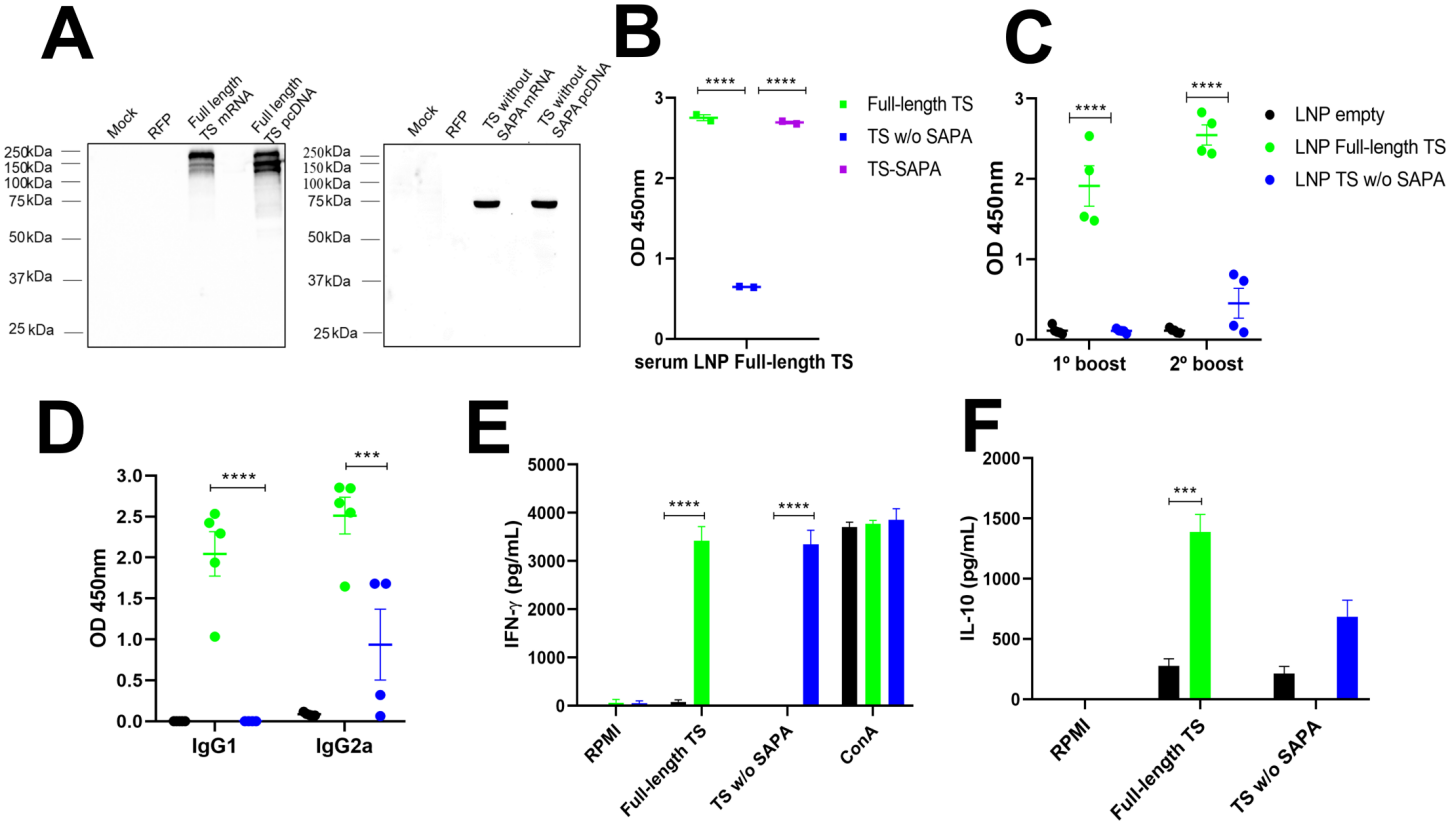
Humoral and cellular responses in mice immunized with RNA encoding TS with or without SAPA. RNA corresponding to the sequences of full-length TS and TS without SAPA repeats was transcribed *in vitro* and used in the immunization protocol. Empty LNPs were used as a control. **(A)** To assess RNA expression, HeLa cells were transfected using lipofectamine and protein expression was confirmed by Western blot using antisera from mice immunized with full-length TS (left) or TS without SAPA (right). Negative controls (Mock and Red Fluorescent Protein - RFP) and a positive control (pcDNA encoding both proteins) are shown in the Western blot. After confirming expression, RNAs were encapsulated into LNPs for use in immunization protocol. Female BALB/c mice were immunized with 10 μg of LNP-formulated RNA following a prime-boost-boost protocol as previously described. **(B)** Immunodominance of SAPA repeats was also observed with RNA formulations. ELISA was performed with serum from mice immunized with LNPs containing full-length TS RNA, which was tested against all three recombinant TS versions. Similar to protein immunization, RNA formulations induced preferential antibody recognition of SAPA repeats over the catalytic domain. ****P < 0.0001. **(C)** Serum was collected after the first and second boosts, and total IgG levels were measured by ELISA using the corresponding recombinant protein for plate coating. ****P < 0.0001. **(D)** IgG1 and IgG2a subclass levels in serum from mice immunized with TS RNA formulations were determined by ELISA. ***P < 0.001, ****P < 0.0001. **(E, F)** IFN-γ **(E)** and IL-10 **(F)** levels were quantified in supernatants from splenocyte cultures incubated with RPMI medium (negative control), stimulated for 72 h with the respective recombinant TS, or concanavalin A (ConA, positive control). ***P < 0.001, ****P < 0.0001.

**Figure 6:**
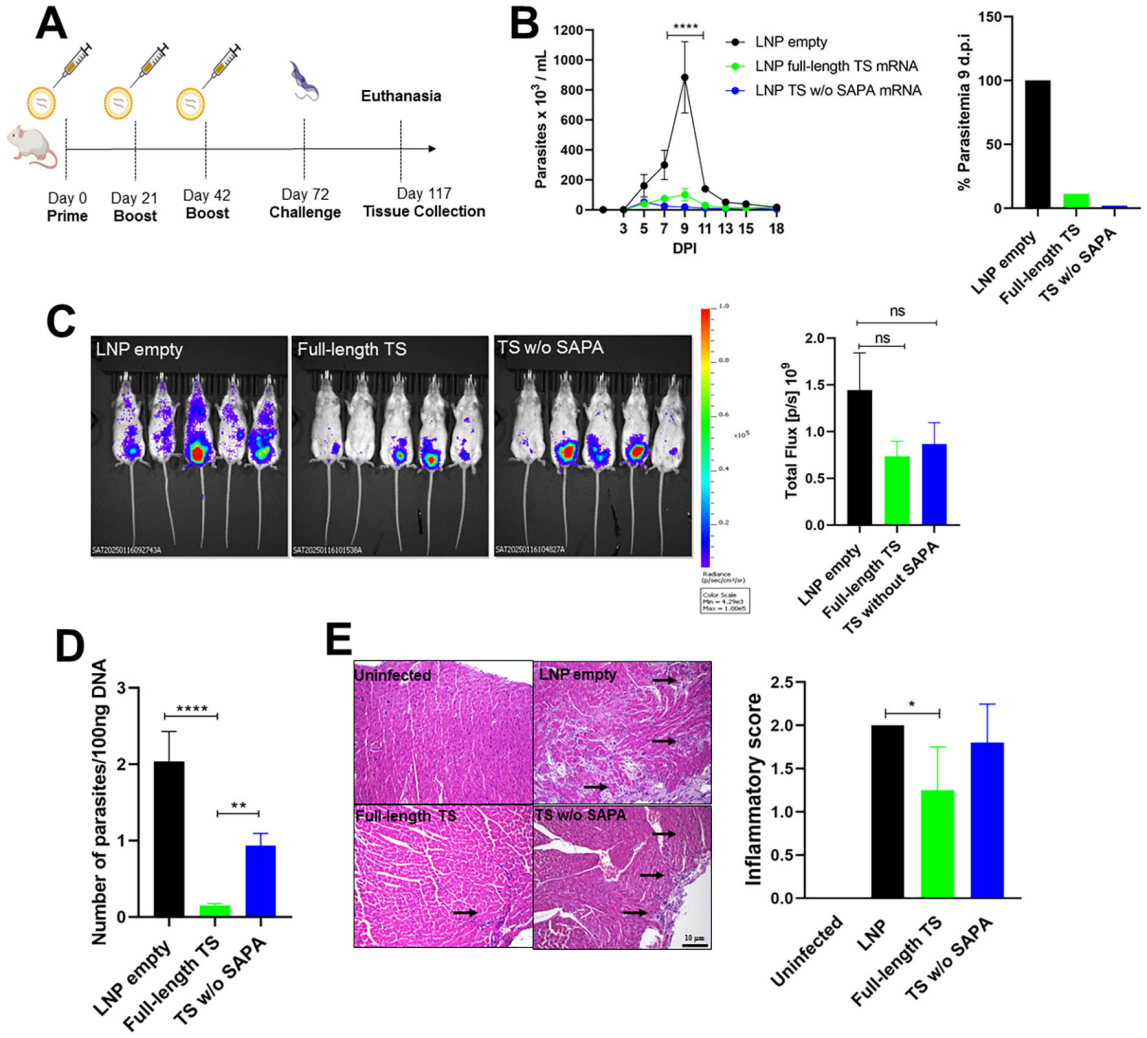
Evaluation of protection from TS RNA immunization following challenge with the *T. cruzi* Y strain. **(A)** Female BALB/c mice were immunized with LNP formulations containing 10 μg of Full-length TS RNA or TS without SAPA RNA using a prime-boost-boost protocol. Thirty days after the last immunization, mice were challenged with 10^4^ bloodstream trypomastigotes of the *T. cruzi* Y strain expressing luciferase. **(B)** Parasitemia in immunized and challenged mice was followed for 18 days (left). The peak of parasitemia was reached at 9 days post-infection (DPI) and presented as a bar graph (right). ****P < 0.0001. **(C)** Tissue parasitism was assessed by bioluminescence using an *In Vivo* Imaging System (IVIS) to visualize luciferase activity from *T. cruzi* in the mice. Bioluminescence quantification is shown on the right. **(D)** qPCR in heart tissue from immunized and challenged animals after 45 DPI. **P < 0.01, ****P < 0.0001. **(E)** Histological analysis of heart tissue showing inflammatory infiltrates (arrows); inflammatory scores are shown in the bar graph (right). *P < 0.05. Scale bar = 10 μm. 20X objective.

The original version of this article has been updated.

