# Supplementary material for: RNA and protein immunization with Trypanosoma cruzi trans-sialidase containing SAPA repeats protects mice against infection and promotes a balanced inflammatory response
Source: Front Cell Infect Microbiol. 2025 Oct 17;15:1681807. doi: 10.3389/fcimb.2025.1681807 (PMC12575306; doi:10.3389/fcimb.2025.1681807)

**
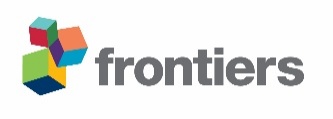
RNA and protein immunization with *Trypanosoma cruzi* trans-sialidase containing SAPA repeats protects mice against infection and promotes a balanced inflammatory response**

**Nailma Silva Aprigio dos Santos^1†^, Carlos Roberto de Almeida-Júnior^1†^, Mayra Fernanda Ricci^1^, Rodrigo C. O. Sanches^1^, Renata Salgado Fernandes^2^, Gabriela de A. Burle-Caldas^2^, Júlia Teixeira de Castro^2^, João Luís Reis-Cunha^3^, Daniella C. Bartholomeu^4^, Ana Clara Martins Meira^1^, Thaiane Gomes Nascimento^1^, Natalia Fernanda de Melo Oliveira^1^, Ricardo T. Gazzinelli^1,2^, Fabiana S. Machado^1^ and Santuza M.R. Teixeira^1,2*^**

Supplementary Material

# Supplementary Data


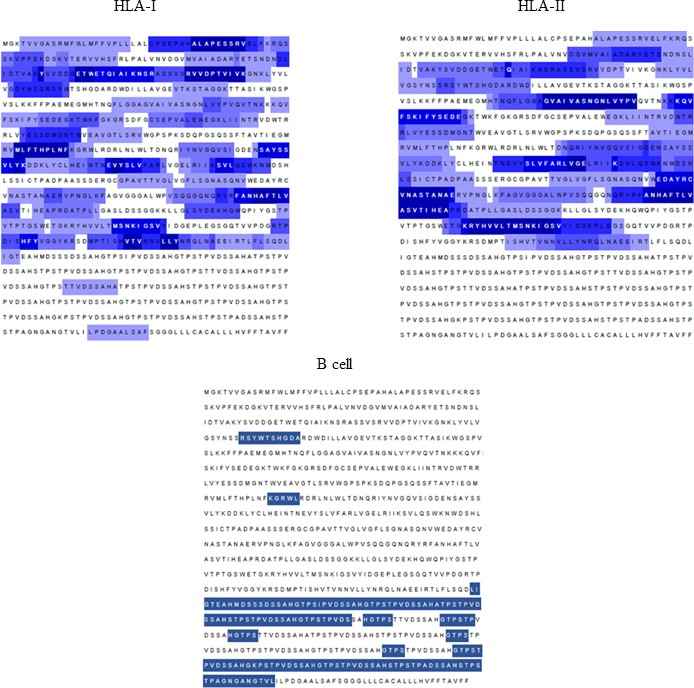
**Supplementary Figure 1:** Sequence analyses for epitope prediction. The sequence used for prediction of CD8⁺ T cell (HLA-I), CD4⁺ T cell (HLA-II), and B cell epitopes.


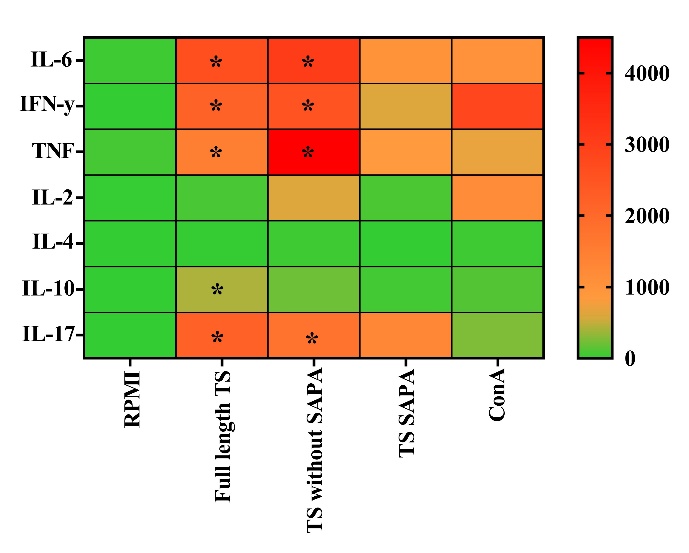
**Supplementary Figure 2**. Cytokine profiling in mice immunized with different recombinant TS versions. Female BALB/c mice were immunized with 10 µg of recombinant TS proteins formulated with alum and CpG adjuvants, using a prime-boost-boost protocol. Thirty days after the last immunization, spleens were harvested for splenocyte culture, and the supernatants were collected for cytokine quantification (IL-6, IFN-γ, TNF, IL-2, IL-4, IL-10, and IL-17) using Cytometric Bead Array (CBA) from mice immunized with full-length TS, TS without SAPA repeats, or TS-SAPA. The RPMI medium (negative control), stimulated for 72 hours with the corresponding recombinant proteins, or concanavalin A (ConA, positive control). **P* < 0.05

**Supplementary Figure 3**. Plasmid used as template for *in vitro* transcription of full-length TS mRNA and TS without SAPA repeats. pcDNA 3.1 vector containing codon-optimized full-length TS **(A)** and TS without SAPA **(B)** repeats sequences flanked by the 5’ and 3’ UTR from human α-1-globulin, and 5’UTR from tobacco virus and 3’ UTR from *Xenopus* β-globin, respectively, and a 110-nucleotide poly-A tail.

**A**


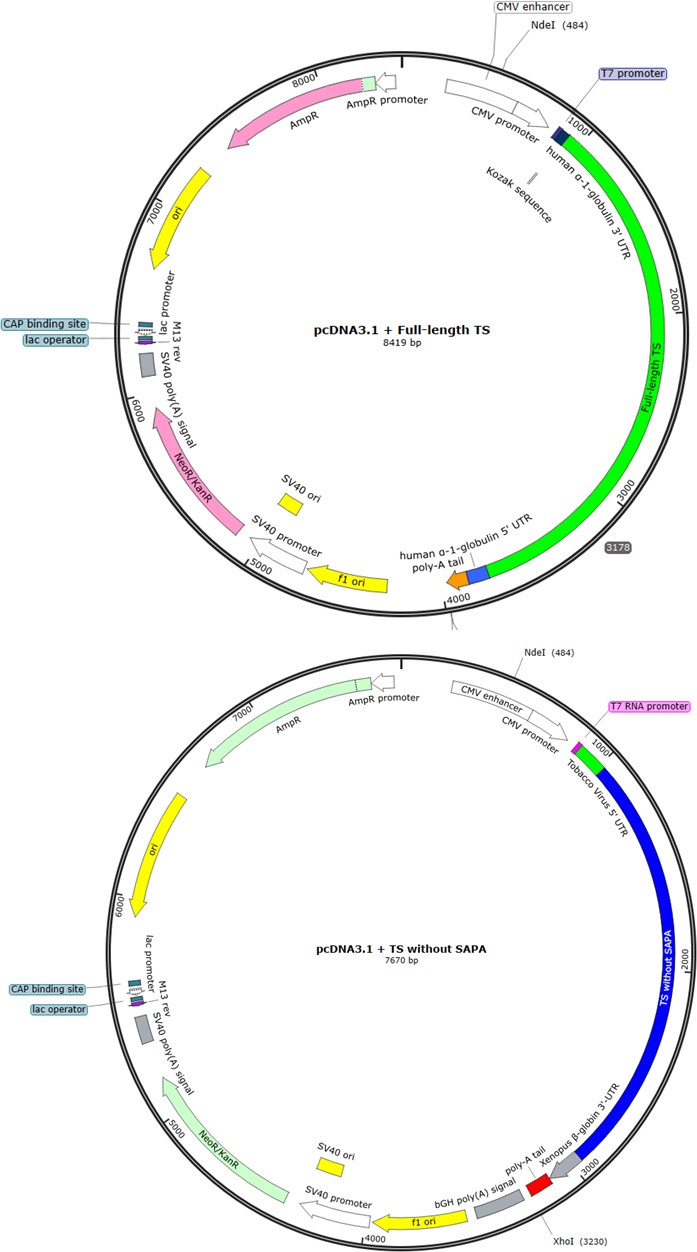


**B**

**Supplementary Table.** mRNA encapsulation efficiency and physicochemical characterization. Mean particle diameter, polydispersity index (PDI), zeta potential, and encapsulation efficiency (%EE) of LNPs formulated with different mRNA constructs.


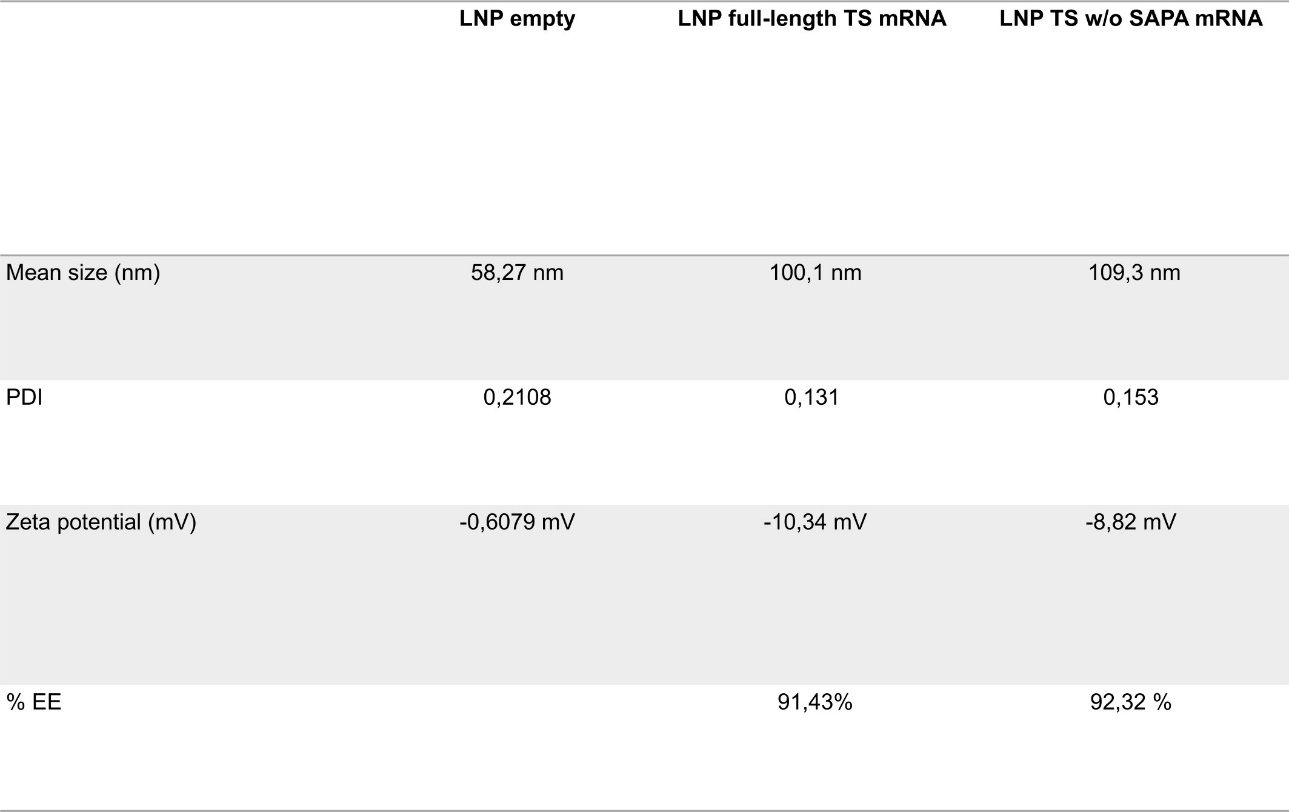

Supplement: Supplementary file 1 [file DataSheet1.docx]
